# Ultrafast infrared nano-imaging of local electron-hole dynamics in CVD-grown single-walled carbon nanotubes

**DOI:** 10.1126/sciadv.adv9584

**Published:** 2025-06-18

**Authors:** Jun Nishida, Keigo Otsuka, Taketoshi Minato, Yuichiro K. Kato, Takashi Kumagai

**Affiliations:** ^1^ Institute for Molecular Science, National Institutes of Natural Sciences, Okazaki, Aichi 444-8585, Japan.; ^2^ The Graduate University for Advanced Studies, SOKENDAI, Hayama, Kanagawa 240-0193, Japan.; ^3^ Nanoscale Quantum Photonics Laboratory, RIKEN Cluster for Pioneering Research, Saitama 351-0198, Japan.; ^4^ Department of Mechanical Engineering, The University of Tokyo, Tokyo 113-8656, Japan.; ^5^ Quantum Optoelectronics Research Team, RIKEN Center for Advanced Photonics, Saitama 351-0198, Japan.

## Abstract

Single-walled carbon nanotubes, as prototypical one-dimensional systems, have been extensively studied for their extreme confinement effects and the formation of strongly bound excitons. However, their high surface-to-volume ratio renders their dynamics highly susceptible to variations in the surrounding environment. Yet, visualizing photoinduced dynamics within individual nanotubes has remained a major challenge because of the lack of methods combining sufficient spatial and temporal resolution with sensitivity to an exceedingly small number of electron-hole pairs. Here, we apply ultrafast infrared nanospectroscopic imaging to probe local electron-hole dynamics in both isolated and bundled carbon nanotubes grown by chemical vapor deposition. This approach unravels heterogeneity in electron-hole pair creation and annihilation, arising from disordered stress within a tube and/or interactions with neighboring tubes. The capability to visualize local electron-hole dynamics in real time and space is essential for advancing carbon nanotubes as fundamental building blocks in nanophotonic and optoelectronic devices.

## Teaser

Ultrafast infrared nano-imaging visualizes local creation and annihilation of electron-hole pairs within carbon nanotubes.

## INTRODUCTION

Single-walled carbon nanotubes (CNTs) exhibit pronounced quantum confinement and reduced dielectric screening effects. Their distinct optoelectronic properties, particularly in semiconducting CNTs, arise from their one-dimensional structure, leading to formation of strongly bound excitons (*1*, *2*), rapid exciton diffusion (*3*–*5*), and pronounced exciton-exciton annihilation (*6*–*11*). These features enable applications such as photosensitizers for harvesting long-wavelength solar energy (*12*), saturable absorbers for ultrafast lasers (*13*), and single-photon sources (*14*, *15*). Their nanoscale dimensions also make them promising for integration in quantum nanophotonics (*16*).

Another key aspect of CNTs, common to low-dimensional materials, is their large surface-to-volume ratio, making them highly sensitive to the surrounding environment. Their optoelectronic response is strongly influenced by substrate interactions (*17*–*19*) and the local dielectric environments (*20*, *21*), which can modify photoluminescence (PL) intensity by orders of magnitude and alter their spectral profiles. In addition, excitons efficiently migrate between neighboring nanotubes (*19*, *22*, *23*), with the precise transport mechanisms remaining under debate.

Given their technological relevance, characterizing the local creation and annihilation of electron-hole pairs is crucial, because these processes govern CNT optoelectronics at the most fundamental level. Optical microscopy techniques, including time-resolved PL microscopy (*24*), transient absorption or nonlinear microscopy (*25*, *26*), and transient interferometric scattering microscopy (*27*), have successfully probed exciton dynamics at the single-CNT levels. However, their spatial resolution is diffraction limited to >200 nm, often insufficient for resolving local disorder within individual CNTs. In contrast, scanning near-field optical microscopy (SNOM) methods, including tip-enhanced Raman (*28*–*30*) and tip-enhanced PL (*30*, *31*) spectroscopy as well as nanolocalized elastic light scattering spectroscopy (*32*), have identified vibrational and electronic disorders (*28*, *32*), chirality switching (*29*), and local defects in CNTs (*30*). Despite their superior spatial resolution (<50 nm) offered by the near-field probe, these techniques primarily offer time-averaged measurements or down-to-nanosecond time resolution (*31*), limiting their use in studying subpicosecond exciton dynamics relevant for, e.g., exciton-exciton annihilation (*10*).

In recent years, ultrafast infrared scattering SNOM (ultrafast IR *s*-SNOM) has enabled probing of local carrier and exciton dynamics with sub–100 nm spatial and subpicosecond temporal resolutions (*33*–*36*). With an expanding range of probe frequencies and enhanced sensitivity, ultrafast IR *s*-SNOM has been widely applied to low-dimensional materials, including monolayer and bilayer transition metal dichalcogenides (*37*, *38*). In addition, ground-state IR *s*-SNOM has also proven useful in observing one-dimensional polaritonic responses in CNTs (*39*, *40*). However, applying ultrafast *s*-SNOM to CNTs remains challenging because of their exceedingly small interaction volumes and the resulting weak transient signals.

Here, we apply ultrafast IR *s*-SNOM to CNTs grown by chemical vapor deposition (CVD) on quartz substrates. By using highly sensitive interferometric detection, we resolve transient mid-IR (MIR) responses from individual CNTs with ~100-nm and ~150-fs spatiotemporal resolutions. We detect photoinduced changes in the near-field scattering amplitude as small as 0.1%, enabling pump-probe measurements of local domains and ultrafast nano-imaging of CNT bundles. We first demonstrate the stability and resolution of ultrafast IR *s*-SNOM in the application to a CNT bundle, revealing pronounced heterogeneity regardless of its topographically uniform appearance. We then show how disordered strain in isolated CNTs leads to nonuniform electron-hole pair creation. Last, we investigate another bundle with spatially evolving tube-tube interactions, which give rise to rich heterogeneity in electron-hole pair annihilation.

In addition, to elucidate the origin of the localized MIR transient signal, we develop a simple theoretical framework modeling a tip apex as a point dipole and a CNT as a segmented one-dimensional dielectric tube. Combined with the analytical estimates of the oscillator strength for intra-excitonic transitions, the model reproduces the observed signal amplitudes and their spatial profiles with a reasonable set of parameters. Our analysis suggests that detecting as few as ~10 electron-hole pairs within a CNT is feasible.

## RESULTS

### Ultrafast IR nano-imaging on CVD-grown CNTs

**Fig. 1.**
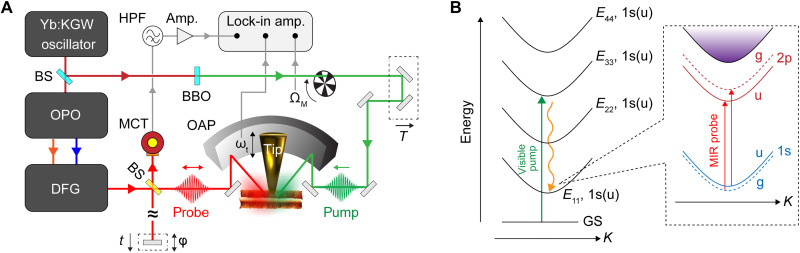
Ultrafast IR nano-imaging of single-walled CNTs. (**A**) Schematic illustration of the ultrafast IR *s*-SNOM setup. BS, beam splitter; BBO, β-BaB_2_O_4_ crystal; OPO, optical parametric oscillator; DFG, difference frequency generation; MCT, mercury-cadmium-telluride detector; OAP, off-axis parabolic mirror; HPF, high-pass filter. (**B**) Schematic energy diagram illustrating optical transitions involved in the measurements, where electron-hole pairs induced by a visible pump pulse are probed by a MIR probe pulse.

Figure 1A shows a schematic illustration of the ultrafast IR *s*-SNOM system (see Materials and Methods for details on the data acquisition scheme as well as notations for the acquired signals) (*38*, *41*). An *s*-polarized visible pump pulse (515 nm/2.4 eV, 60 to 250 μJ/cm^2^) is focused onto CNTs or their bundles to generate electron-hole pairs, followed by a *p*-polarized MIR probe pulse (6 μm/0.2 eV, <1 μJ/cm^2^). For semiconducting CNTs, the density of states is characterized by van Hove singularities, and given the average diameter of our CNTs of 1.3 to 1.4 nm, the 2.4-eV pump pulse is most likely resonant with their *E*_33_ transition (*42*). The sample also contains nanotubes with larger diameters (~1.8 nm), for which the excitation is instead more closely resonant with the *E*_44_ transition. The population at the higher energy level is believed to be short lived (<50 fs) (*43*), relaxing to the *E*_11_ state within our time resolution of ~150 fs. Electron-hole pairs created in the *E*_11_ state result in the MIR transient signal, the detailed mechanism of which will be discussed in a later part. In Fig. 1B, we depict intra-excitonic transition from the 1s to 2p state as a potential underlying contribution, with each state splitting into even (g) and odd (u) parity levels (*44*, *45*).

**Fig. 2.**
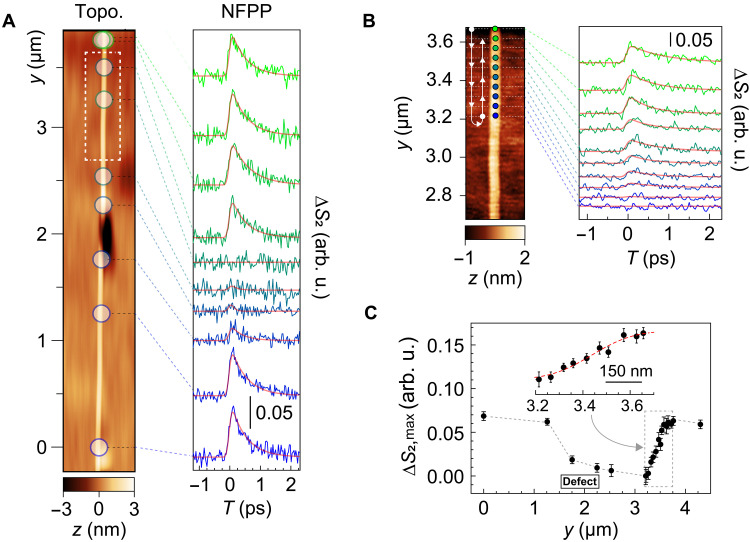
Local pump-probe measurements of a CNT bundle. (**A**) AFM topography (left) and spatially resolved near-field pump-probe (NFPP) signals (Δ*S*_2_, right) of CVD-grown CNTs. The pump and probe wavelengths are 515 nm and 6 μm, respectively. (**B**) High-resolution pump-probe scan of a white dotted region in (A). Arrows in the topography indicate the sequence of the pump-probe measurements. (**C**) Spatial evolution of the peak value of the pump-probe signal (Δ*S*_2,max_) at different locations. Error bars represent the 1σ standard deviation obtained from fitting the temporal profiles to an exponential decay function.

We first demonstrate the capability and stability of ultrafast IR nano-imaging to resolve dynamical heterogeneity within CNTs. The CNT samples, grown by CVD on r-cut quartz substrates, consist of a mixture of different chirality, specified by the chiral index (*n*, *m*), and include both isolated and bundled CNTs (see Supplementary Note 1 for sample preparation details). Figure 2A (left) shows the atomic force microscope (AFM) topography of a representative CNT bundle. While the bundle appears as a single tube in the AFM image, its topographic height (~3 nm) exceeds the expected diameter of individual CNTs in the sample, indicating that the structure comprises two or three tubes. We acquired pump-probe relaxation profiles at 10 selected locations marked by circles, and the corresponding pump-probe signals are shown in Fig. 2A (right). Regardless of the homogeneous appearance of the bundle in the AFM topography, the transient MIR signals exhibit pronounced heterogeneity. A pump-probe signal is observed clearly at the bottom of the tube (*y* < 1.5 μm), but it is suppressed near a substrate defect located at *y* ≈ 2 μm. Beyond this defect, the absence of the signal persists up to *y* ≈ 3.2 μm. Notably, between *y* = 3.2 and 3.5 μm, the pump-probe signal recovers to the original level despite the absence of any corresponding features in the AFM topography within the transition region.

To further investigate this transition region at *y* = 3.2 to 3.5 μm, we performed pump-probe measurements with a finer step of 50 nm (Fig. 2B). In spite of the apparent topographic uniformity, the near-field pump-probe signal exhibits substantial variation, consistent with observations in Fig. 2A. The measurements were conducted in an alternating order (indicated by arrows in Fig. 2B, left), ruling out artifacts such as gradual photodegradation of the CNTs or the AFM tip. By combining data in Fig. 2 (A and B), we plot the maximum of the pump-probe amplitudes across the bundle (Fig. 2C), derived from single-exponential fitting to each decay curve. These data reveal that the substrate defect at *y* ≈ 2 μm largely disrupts electron-hole pair formation in the bundle, with the suppression propagating over a distance >1 μm, even without direct contact between the defect and the CNTs. On the basis of the ultrafast nano-imaging data, we estimate the spatial resolution of the measurement to be ~130-nm full width at half maximum (FWHM) (fig. S1). After deconvolving the data with this resolution, the transition behavior observed in the *y* = 3.2- to 3.5-μm region (Fig. 2C, inset) is fit well by an error function with a width of ~200-nm FWHM. This reflects a sharp yet finite-size domain over which the self-correction of the electron-hole pair formation occurs. The observed heterogeneity in Fig. 2 (A to C) underscores the necessity to perform ultrafast nano-imaging to gain a comprehensive understanding of the local creation of electron-hole pairs. Furthermore, the stability and precision of the measurement are crucial to unravel detailed structural and dynamical heterogeneity.

### Correlation between dynamical heterogeneity and disordered lattice strain

We now explore the microscopic origin of the dynamical heterogeneity observed in Fig. 2. Previous studies suggest that chirality switching in CNTs during growth can alter their electronic structure (*29*). However, chirality switching is rare in CVD-grown CNTs, occurring approximately once per millimeter (*46*, *47*), which is inconsistent with the domain switching observed here, occurring twice within ~1 μm segment. Furthermore, previously reported chirality switching occurs over short length scales (<100 nm) (*29*), whereas the switching domain in Fig. 2 (B and C) spans a longer region. These discrepancies indicate that chirality switching is unlikely to account for the heterogeneity in electron-hole pair formation observed here.

Instead, a substrate defect appears to play a dominant role, underscoring the importance of CNT-substrate interactions. Because chirality switching occurs independently of the substrate, it is again unlikely to be responsible for the observed variations. CVD-grown CNTs undergo compressive stress during postgrowth cooling because of the thermal expansion mismatch with the substrate. Surface defects and roughness then introduce disordered compressive strain along the CNT axis (*48*), which likely contributes to the spatial nonuniformity in the pump-probe signal.

**Fig. 3.**
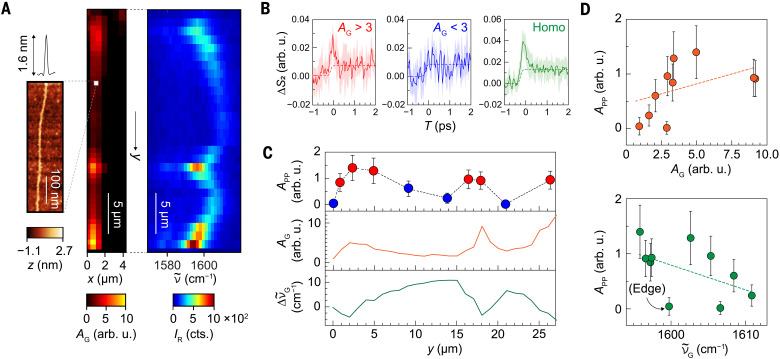
Strain-correlated disorder in a single and isolated CNT. (**A**) High-resolution AFM topography image (left) obtained by peak-force-tapping mode with a sharp SiN tip, integrated G-band area *A*_G_ from Raman microscopy (center), and the corresponding evolution of the G-band spectra (right) for a single and isolated CNT. (**B**) Averaged pump-probe signals from a less compressive domain (red) and a compressive domain (blue), together with an averaged pump-probe signal on another CNT with homogeneous G-band intensity (green). The pump and probe wavelengths are 515 nm and 6 μm, respectively. The shaded regions represent the standard deviation (1σ) of the pump-probe signal, calculated from *n* ≥ 4 samples. (**C**) Spatial variation of the pump-probe signal amplitude (*A*_pp_, top), G-band peak area (*A*_G_, middle), and G-band peak position shift ( $∆{\tilde{\nu }}_{G}={\tilde{\nu }}_{G}-1600\;{\text{cm}}^{-1}$ , bottom) at the different locations within the CNT. (**D**) Correlation plots of *A*_pp_ versus *A*_G_ (top) and *A*_pp_ versus ${\tilde{\nu }}_{G}$ (bottom). Error bars in (C) and (D) represent the 1σ SD.

To investigate the relationship between lattice strain disorder and electron-hole pair dynamics, we performed correlative nanoscale pump-probe measurements and Raman microscopy, with the latter being highly sensitive to local lattice strain. A single, isolated CNT (~30 μm long) was selected to eliminate the complexity associated with tube-tube interactions despite the lower signal level. Radial breathing mode (~140 cm^−1^) and resonance Raman effect with a 532-nm excitation identified the CNT as a (17,9) semiconducting nanotube with a 1.8-nm diameter. High-resolution AFM imaging (see Materials and Methods for details) confirmed a ~1.6-nm topographic height (Fig. 3A, left), consistent with an isolated tube.

The Raman G-band (1580 to 1620 cm^−1^) mapping in Fig. 3A (center and right panels), presented as integrated intensity *A*_G_ and corresponding spectral profiles, reveals the discernible variations along the CNT axis, reflecting local strain disorder. In stress-free CNTs, the G-band peak position ${\tilde{\nu }}_{G}$ appears at ~1590 cm^−1^, while compressive strain shifts it to higher frequencies (*48*). The correlation between the G-band peak position ${\tilde{\nu }}_{G}$ and the intensity *A*_G_ likely arises from the strain-induced modulation of the CNT band structure. For the (17,9) CNT, compressive strain reduces the *E*_44_ transition energy (*49*, *50*), affecting conditions for the resonance Raman effect and, thus, the enhancement factor.

Local pump-probe measurements at 10 different locations within the CNT reveal a clear dependence on strain. As shown in Fig. 3B, the average pump-probe signal from regions with low compressive strain (six points with high G-band intensity, *A*_G_ > 3, red) exhibits a distinct rise-and-decay feature, whereas in strongly compressed regions (four points with *A*_G_ < 3, blue), such features are suppressed. In addition, we performed a separate measurement on another CNT with a homogeneous G-band intensity profile (Supplementary Note 4 and fig. S3), which yielded a clearer rise-and-decay feature (Fig. 3B, green). The long-lived offsets were observed in a metallic tube or even on a substrate, suggesting that this is an artifact pump-probe signal arising from the tip itself (fig. S3). Note that this tip-originating signal is only evident under a strong excitation to detect an exceedingly weak signal and thus is negligible for most applications of ultrafast IR *s*-SNOM. The differing pump-probe responses in weakly and strongly stressed regions (Fig. 3B, red and blue) highlight the impact of local strain on photoinduced electron-hole pair dynamics.

The correlation between local compressive strain and pump-probe signal is further demonstrated by plotting the pump-probe amplitude along the CNT axis (Fig. 3C, top), alongside corresponding G-band Raman intensity (middle) and peak shift (bottom). The spatial evolution of pump-probe signals aligns closely with Raman behavior, as supported by correlation plots (Fig. 3D). One outlier data point originates from the edge of the CNT, where the exciton quenching at the edge states may influence the signal (*51*). Deviations from perfect correlation may also arise from limited spatial alignment accuracy between the two measurements (~1 to 2 μm), as well as differences in excitation wavelengths (532 nm for Raman and 515 nm for ultrafast IR *s*-SNOM). Despite these uncertainties, the correlation suggests that disordered strain along the CNT axis drives heterogeneity in electron-hole pair creation.

The correlations between the pump-probe amplitudes and Raman parameters also likely stem from the strain-induced modulation of the band structure. Compressive strain reduces the *E*_44_ transition energy and enhances the mismatch between the absorption resonance (~532 nm unstrained) and the 515-nm excitation, leading to less efficient pumping in strained regions. A simple model in Supplementary Note 9 predicts a nearly linear relationship between pump-probe amplitude and G-band intensity, as well as between pump-probe amplitude and compressive strain. Deviations from linearity in Fig. 3D may be due to the limited signal-to-noise ratio or unaccounted effects such as saturation effects (fig. S5), strain-induced modulation of the *E*_44_ transition oscillator strength, or nonunimodal excitation-energy dependence of the resonance Raman enhancement factor (*52*).

We note that the extraction of the annihilation time constants for the single CNTs was hindered by the limited signal-to-noise ratio. Yet, the data from the bundle in Fig. 2, which are also likely influenced by strain modulation, demonstrate minor variation in relaxation dynamics despite substantial changes in the corresponding amplitudes. Given the weak dependence of the annihilation dynamics on the excitation fluence (fig. S5C), this behavior is also consistent with the strain-induced tuning of the band structure as envisioned above.

These visible-pump and IR-probe measurements, performed at the single-CNT level, offer a means to understand the origin of the MIR transient signal. By comparing pump-probe signal amplitudes (Δ*S*_2_) in Fig. 3B to the simultaneously acquired ground-state scattering signal (*S*_3_), a ratio of Δ*S*_2_/*S*_3_ ~ 0.12% is estimated, which is quantitatively analyzed in a later section to relate the transient MIR response to intra-excitonic transitions.

We note that a high pump fluence (250 μJ/cm^2^ or 6 × 10^14^ photons pulse^−1^/cm^2^) was used for the measurement above. This fluence is close to the threshold (5 × 10^14^ photons pulse^−1^/cm^2^) where pulsed excitation can alter CNT PL profile because of the formation of laser-induced defects and dark-exciton activation from symmetry breaking (*53*). The exact threshold likely depends on synthesis methods and local CNT environments. To address the possibility of laser-induced heterogeneity, we performed a similar measurement on another CNT with a lower excitation fluence (60 μJ/cm^2^ or 1.5 × 10^14^ photons pulse^−1^/cm^2^), yielding similar correlations between strain and MIR transients (Supplementary Note 4 and fig. S4). This supports that the observed heterogeneity originates from intrinsic local strain rather than laser-induced effects.

### Dynamical heterogeneity in CNT bundles

**Fig. 4.**
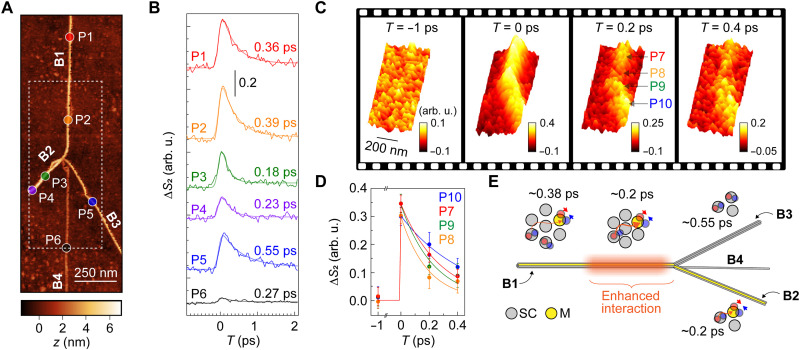
Ultrafast IR nano-imaging of CNT bundles. (**A**) Peak-force-tapping AFM image of CNT bundles, showing a bundle originating from the top that splits into multiple smaller bundles at the center of the image. (**B**) Local pump-probe signals observed at different locations within the bundle, with time constants extracted from single-exponential fits. The pump and probe wavelengths are 515 nm and 6 μm, respectively. (**C**) Ultrafast IR nano-imaging acquired within the dotted square domain in (A). (**D**) Pump-probe decay curves at different locations, as extracted from the imaging in (C). (**E**) Proposed mechanism of the heterogeneous annihilation, where metallic CNTs act as quenching sites and exciton transfer into these sites plays a critical role. SC, semiconducting CNT; M, metallic CNT.

In CNT bundles consisting of multiple and closely packed tubes, the interactions among the tubes are expected to play critical roles in the dynamics of the photoinduced electron-hole pairs. In Fig. 4A, we show a high-resolution peak-force-tapping AFM image of one such bundle: a primary bundle (B1) originating from the top of the figure branches into multiple bundles (B2, B3, and B4) around the center of the image. The larger AFM topographic height of ~6 nm, in comparison to the isolated CNT in Fig. 3A, confirms that this bundle is composed of multiple CNTs. We first performed local pump-probe measurements, and the results are plotted in Fig. 4B. The pump-probe profiles reveal heterogeneity not only in the signal amplitudes but also in the relaxation dynamics, as characterized by single-exponential fits to the decay. The highest signal amplitudes were observed at points P1 and P2 in the original branch B1. The signal splits into the lower levels as the bundle branches, with nontrivial evolution in the annihilation dynamics. Point P5 in the B3 branch exhibits markedly slower decay (0.55 ps) compared to points in the B1 branch (0.36/0.39 ps), while points P3 and P4 in the B2 branch (0.23 and 0.18 ps) and point P6 in the B4 branch (0.27 ps) show faster decay.

To visualize dynamical heterogeneity in real space and time, we performed ultrafast IR *s*-SNOM imaging within the white dotted region in Fig. 4A. The results, presented in Fig. 4C, quantitatively reproduce the behavior inferred from the local pump-probe signals in Fig. 4B. At *T* = 0 ps, the signal in the B1 branch is slightly higher than in the B3 branch. In contrast, at *T* = 0.4 ps, the B3 branch exhibits a larger signal than the B1 branch, indicating slower annihilation dynamics in the B3 branch compared to the B1 branch, consistent with the local pump-probe measurements in Fig. 4B. The pump-probe signal in the B2 branch is barely visible in the image, while the B4 branch is obscured by noise because of the shorter averaging time used for imaging compared to the local pump-probe decay measurements. In the time-resolved imaging, point P8 located at the onset of the B1 branch splitting into multiple branches, the annihilation is markedly faster than in other regions. We extracted pump-probe decay profiles from the imaging as shown in Fig. 4D, which are in good agreement with the results in Fig. 4B within errors. The key trend of slower annihilation in the B3 branch (at point P10) compared to the B1 branch (at point P7) is consistently reproduced, further underscoring the reliability and quantitative capability of ultrafast IR nano-imaging for probing electron-hole dynamics within CNTs.

We now address the microscopic origin of the observed dynamical heterogeneity in the CNT bundle. Electron-hole pairs generated in neighboring CNTs are known to undergo excitation transfer, with the transfer times as fast as 100 to 500 fs (*22*, *23*). Here, the bundle consists of CNTs with various chiralities (*n*, *m*), including not only semiconducting CNTs as a source of the transient MIR signal but also metallic CNTs for which *n* − *m* ≡ 0 (mod 3). The electron-hole pairs transferred to metallic tubes are rapidly quenched because of the absence of the bandgap (*54*). Therefore, one plausible explanation for the observed dynamical heterogeneity is that excitation transfer from semiconducting CNTs to metallic CNTs governs the annihilation process within the bundle (Fig. 4E), as previously suggested (*54*). This mechanism explains why the lifetime in the original B1 branch is approximately an average of those in B2 and B3; upon branching from B1, a higher fraction of metallic CNTs enters B2 compared to B3. This results in faster decay in B2 and a prolonged lifetime in B3, as the latter contains fewer metallic CNTs acting as quenching sites. In addition, the extraordinarily enhanced annihilation at point P8 in the B1 branch suggests that even with an identical bundle composition, subtle variations in tube-to-tube distance and orientation can have a large impact on excitation transfer rates.

This mechanism, where the annihilation of electron-hole pairs in the bundle is governed by quenching in metallic tubes, is further supported by the bundle height dependence of the amplitudes and decays of the pump-probe signals (Supplementary Note 7 and fig. S7). Despite the limited statistics, measurements across various tube structures within the sample demonstrate that the pump-probe signal saturates as the topographic height of the bundle increases. In addition, the relaxation lifetime peaks at a certain height and then decreases with further increases in height. These behaviors may be explained by the increasing probability of containing metallic tubes in a larger bundle; as the bundle grows in size, the inclusion of more metallic CNTs likely enhances quenching, reducing the overall signal amplitude and facilitating the recombination rates. These findings collectively highlight the complex interplay between chirality, structural heterogeneity, and excitation transfer in determining the electron-hole pair behavior in CNT bundles.

We note that the ultrafast IR nano-imaging in Fig. 4 was performed with a high fluence of ~250 μJ/cm^2^. In Supplementary Note 10 and fig. S10, we demonstrate that ultrafast IR nano-imaging on a bundle remains feasible with the excitation fluence down to ~30 μJ/cm^2^ while maintaining the capability of resolving heterogeneity within the bundle.

### Origin of the nanoscale transient MIR response

Here, we explore the microscopic mechanisms behind the transient MIR response in CNTs and how it manifests in near-field scattering measurements. In semiconducting CNTs, photoinduced electron-hole pairs are believed to primarily exist as bound excitons (*1*, *2*) rather than free carriers. While a Mott transition, i.e., exciton dissociation into electron-hole plasma under high excitation fluence, has been observed in two-dimensional transition metal dichalcogenides (*37*, *55*, *56*), it remains difficult to achieve in one-dimensional CNTs. Instead, exciton density saturates before reaching the Mott threshold, as supported by fluence-dependent PL (*57*) and pump-probe spectroscopy (*58*). Consistently, our measurements show signal saturation at high pump fluences (Supplementary Note 5 and fig. S5).

**Fig. 5.**
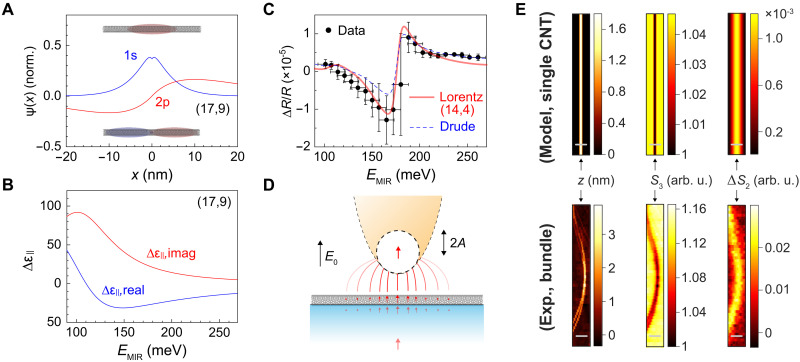
Simulation of ultrafast IR transient response and nano-imaging on CNTs. (**A**) Analytically calculated wave functions of bound electron-hole pairs in a (17,9) semiconducting CNT. (**B**) Calculated real and imaginary parts of the photoinduced change of the on-axis dielectric function (Δε_||_) arising from the 1s-2p transition, assuming the wave function in (A) and an exciton density of 1 × 10^6^ cm^−1^. (**C**) Far-field ensemble-averaged reflection pump-probe spectrum (dots) with 1σ sigma error bars from four independent measurements. Red line: fit using the scaled dielectric function from (B); blue dotted line: fit using a divergent Drude response. (**D**) Schematic illustration of near-field interactions between the CNT, modeled as a segmented dielectric cylinder, and the tip apex, modeled as a point dipole. (**E**) Comparison between experimental and simulated ultrafast IR nano-imaging. Top: simulated topography (*z*, left), ground-state IR *s*-SNOM (*S*_3_, middle), and pump-probe IR *s*-SNOM (Δ*S*_2_, right) of a single (17,9) CNT. Bottom: experimental topography (*z*, left), ground-state IR *s*-SNOM (*S*_3_, middle), and pump-probe IR *s*-SNOM (Δ*S*_2_, right) of a CNT bundle. Scale bars, 150 nm.

Given the saturation behavior, we examine how the highest possible exciton density contributes to the transient MIR response. Previous visible-pump IR-probe studies on CNTs attribute this response to intra-excitonic 1s-2p transitions (*44*, *59*). In bulk semiconductors, these transitions typically occur in the terahertz range at low temperatures (*60*), but in low-dimensional materials with strong exciton binding energies, they can shift into the MIR range (*61*–*63*). Using a model that treats excitonic wave functions on a dielectric cylinder (*44*, *45*, *64*), we estimate the contribution of the 1s-2p intraexcitonic transition in a (17,9) CNT that is discussed in Fig. 3. Some of the simulation parameters, including effective dielectric screening and damping rate, were chosen to reproduce the ensemble-averaged far-field pump-probe spectrum in Fig. 5C. The effective reduced mass of the electron-hole pair was inferred on the basis of the chirality (*65*). See Supplementary Notes 2 and 8 for the details on the modeling and comparison to the far-field pump-probe spectrum.

The simulated excitonic wave functions (Fig. 5A) reveal the electron-hole separation of ~3.25 nm in the 1s state, in agreement with the expected Bohr radius for the relatively large (17,9) CNTs (*66*). The exciton binding energy is 125 meV, and the 1s-2p transition energy is 110 meV. At high pump fluence, we assume that the exciton density reaches saturation constrained by an interexciton spacing of ~10 nm, corresponding to a multiple of the mean exciton-hole separation length to fully exclude the overlap of the 1s wave function among neighboring excitons. This yields a saturation density of 1 × 10^6^ cm^−1^, aligning with a prior study (*57*). Note that the saturation density observed in PL measurements is typically much lower because of contributions from exciton diffusion over tens of picoseconds.

The computed photoinduced change in the dielectric function along the CNT axis (Δε_||_) is shown in Fig. 5B, demonstrating a substantial response given the small intrinsic MIR dielectric function (~5) of CNTs (*67*). To validate this model, we compare the far-field ensemble-averaged reflection pump-probe spectrum with a calculated response for a (14,4) CNT, which has a similar diameter to the average of the diameters (1.3 to 1.4 nm) in our sample. The resulting dielectric function, scaled by exciton density to account for the sparse CNT distribution and lower excitation fluence, reproduces the ensemble-averaged reflection pump-probe spectrum reasonably well (Fig. 5C). The strongly dispersive feature arises from the Si_O phonon resonance of the quartz substrate (*68*), which is considered in the multilayer reflection simulation in the three-layer system (air, CNT, and quartz; see Supplementary Notes 1 and 6 for details). The modeling with the intra-excitonic transition (transparent red line) reproduces the observed features slightly more accurately than a fitting with a divergent Drude response (blue dotted line).

For near-field measurements, the pump-probe signal is typically dominated by Δε_⊥_, the transient dielectric response perpendicular to both the substrate surface and the CNT axis. In a homogeneous environment, this component would be zero because of the cylindrical symmetry of the excitonic wave function. However, the presence of the substrate breaks the symmetry, inducing out-of-plane polarization and making Δε_⊥_ nonzero. In addition, because the tip axis is not perfectly aligned with the surface normal, some in-plane polarization may inherently contribute to the pump-probe signal. To account for these effects, we introduce a phenomenological geometric factor *f*_g_ to relate Δε_||_ to Δε_⊥_ (Δε_⊥_ = *f*_g_ × Δε_||_, 0 < *f*_g_ *<* 1) and use *f*_g_ as the sole fitting parameter to match the calculated pump-probe amplitude to the experimentally observed one for the (17,9) tube in Fig. 3.

### Near-field interactions for one-dimensional systems in IR *s*-SNOM

IR *s*-SNOM is a measurement that essentially probes the dielectric functions of a material underneath a metallic tip. With the duration of the pump/probe pulses (~150 fs) far exceeding the period of the MIR field oscillation (~20 fs), ultrafast IR *s*-SNOM signals are closely related to the photoinduced change in the dielectric function. The relationship of the material dielectric function to the scattering profile observed in IR *s*-SNOM has been extensively studied for various configurations, and many approaches have been proposed (*69*, *70*). Among them, the point dipole model effectively reproduces a nano-Fourier transform IR spectral phase response within an accuracy of a factor of 2 (*69*, *71*) despite its extreme simplicity. Using this approach, we analyze the near-field interactions between the tip and a CNT, modeled as a dielectric cylinder, and explore how the polarization induced in the tip-CNT-substrate system depends on the tip-sample distance (Fig. 5D). The full details are provided in Supplementary Note 2.

In this model, the optical response of the tip is represented as a dipole at the center of a metallic sphere, with an effective radius of 200 nm to reproduce the spatial localization of the experimental pump-probe signal (~130-nm FWHM; Fig. 5E). The CNT, being much smaller than the tip apex, experiences a locally homogeneous electric field. For a dielectric cylinder in a uniform field, the induced polarization line density per unit length (*p*_d_) is expressed as

$$p_{d}=2\pi \varepsilon_{0}r_{\text{CNT}}^{2}\beta_{\text{CNT}}E_{\text{ext}}$$ (1)

where ε_0_ is the vacuum permittivity, *r*_CNT_ is the radius of the CNT, and $\beta_{\text{CNT}}=\left(\varepsilon_{\text{CNT}}-1\right)/\left(\varepsilon_{\text{CNT}}+1\right)$ contains the dielectric function ε_CNT_ of the CNT (see Supplementary Note 2 for the derivation of Eq. 1). We segment the CNT into *N* small fractions and seek self-consistent solutions that account for the interactions among the CNT dipoles, the tip dipole, and their mirror dipoles within the substrate. The polarization response is computed for harmonically modulated tip-sample distance, the demodulation of which is directly comparable to the experimental data. The near-field pump-probe signals are calculated as the difference between the demodulated responses computed using the ground-state (*67*) and excited-state dielectric functions of the CNT. We analyze the second harmonic pump-probe signal Δ*S*_2_ and the third-harmonic ground-state signal *S*_3,gs_, both of which are experimentally sufficiently localized with a good signal-to-noise ratio.

In Fig. 5E (top), we present the simulated AFM topography, ground-state profile *S*_3_, and pump-probe profile Δ*S*_2_, calculated using the dielectric function in Fig. 5B and a phenomenological geometrical factor *f*_g_ set to 0.048. These parameters reproduce the experimentally observed pump-probe signal amplitude of Δ*S*_2_/*S*_3_ ≈ 0.12% in the (17,9) CNT. The relatively small geometrical factor aligns with the expectation that Δε_⊥_ is weaker than Δε_||_. This successful reproduction supports the intra-excitonic 1s-2p transition as a viable mechanism for the observed nanoscale pump-probe signal.

The calculated pump-probe signal profile Δ*S*_2_ is spatially more extended than the “darkening” of the ground-state signal *S*_3_, which is localized to the AFM topographic profile. We note that higher-order demodulation does not substantially enhance the localization of the pump-probe signal in this case because of the small dielectric function of the substrate (Supplementary Note 3 and fig. S2). These contrasting behaviors between *S*_3_ and Δ*S*_2_ are experimentally corroborated as shown in Fig. 5E (bottom) for a CNT bundle; the dip in the ground-state signal *S*_3_ closely follows the topography, while the pump-probe signal Δ*S*_2_ extends beyond it (~130-nm FWHM). This observation highlights the distinct mechanisms underlying the ground-state and pump-probe *s*-SNOM signals. The darkening in the ground-state signal arises from topographic effects, where the tip scanning over the CNT creates an effective void between the tip apex and the substrate, reducing the overall near-field interaction (*72*). In contrast, pump-probe *s*-SNOM, which detects the photoinduced “change” in the dielectric function, is largely immune to the topographic artifacts in the current configuration. Its spatial profile is governed primarily by pure optical contrast, rendering ultrafast IR nano-imaging a robust tool for probing photoinduced optical responses.

## DISCUSSION

The annihilation rates of electron-hole pairs in CNTs have been extensively studied, with reported lifetimes ranging from subpicosecond to tens of picoseconds (*14*, *24*, *27*). The rapid decay rates observed in this study (<1 ps) (*22*, *44*) are among the shortest reported and likely arise from three potential mechanisms, namely exciton-exciton annihilation, substrate-induced dissipation, and above-bandgap excitation.

First, the high excitation fluence (>60 μJ/cm^2^) leads to strong exciton-exciton annihilation. The saturation behavior in the fluence-dependent near-field pump-probe measurements (fig. S5 and Supplementary Note 5) signifies the strong interactions among excitons. The assumed exciton density (~1 × 10^6^ cm^−1^) corresponds to an average spacing of ~10 nm per exciton. Excitons in CNTs diffuse over 100 nm in less than 100 ps (*3*, *57*), and because of the time-dependent diffusion length of $\sqrt{2\mathrm{Dt}}$ , they can travel ~10 nm within 1 ps, rapidly encountering other excitons and annihilating in a reaction-limited manner (*10*). The observed rapid quenching thus suggests that the spatial profiles at scales above 100 nm (Figs. 2 to 4) do not involve the effect of diffusion, in contrast to previous observations in, e.g., tip-enhanced PL measurements (*51*). Furthermore, far-field pump-probe measurements with a lower excitation fluence (<1 μJ/cm^2^) exhibit distinctively slower relaxation (Supplementary Note 6 and fig. S6), corroborating the role of high exciton densities in the observed near-field pump-probe decays.

Second, exciton dynamics are also strongly influenced by the underlying substrate. CNTs grown directly on quartz exhibit much weaker PL compared to those on hexagonal boron nitride, indicating enhanced nonradiative decay on quartz (*18*). Even at the low fluence below 1 μJ/cm^2^ used in our far-field pump-probe measurements, the observed decay time of 1 to 2 ps is much shorter than the 50- to 100-ps lifetimes of fully isolated CNTs (*24*), suggesting substantial energy dissipation into the substrate. While the exact mechanism remains uncertain, a quartz substrate demonstrates particularly detrimental effects on the PL emission of CNTs compared to other dielectric substrates (*19*). The highly polar nature of the quartz substrate may facilitate exciton localization, leading to rapid relaxation (*73*). In addition, the high-frequency phonon modes of the quartz may contribute to and accelerate multiphonon decay processes (*73*).

Last, above-bandgap excitation may further contribute to rapid decay. Under such conditions, carriers initially populate higher energy states (*E*_33_ or *E*_44_) before relaxing to the lowest energy state (*E*_11_), generating a substantial phonon population. Given the critical role of exciton-phonon coupling in CNTs (*74*), this hot-phonon effect likely affects exciton relaxation, even at lower excitation fluences.

While the transient MIR signal is attributed to intra-excitonic transitions in the discussion above, contributions from free carriers cannot be entirely ruled out. Although excitons typically dominate CNT optical properties (*1*, *2*, *57*), some studies report excitation-induced terahertz conductivity changes suggestive of free carriers (*75*, *76*), while multiterahertz spectroscopy favors an excitonic picture (*77*). Our far-field reflection pump-probe spectrum is slightly better fit with the excitonic model rather than Drude free carrier models (Fig. 5C). In addition, near-field pump-probe signal amplitudes are reasonably reproduced with intra-excitonic transitions as we delineated in the theoretical analysis. Yet, we note that our modeling is only semiquantitative because of the lack of consideration of the elongated tip geometry (*70*), in addition to uncertainties in parameters such as dephasing rate, geometric factor *f*_g_, or exciton density.

Nevertheless, the successful reproduction of the transient signal using a plausible set of parameters supports the finding that the exciton model adequately captures our observations without assuming the presence of free carriers in CNTs. Although intentional doping or external voltage exertion previously resulted in inducing trions and free carriers in CNTs (*78*, *79*), our neutral CNTs with a minimal amount of defects (*11*, *80*) suppress these contributions. Assuming that the observed electron-hole pairs exist as bound excitons with their density close to the saturation density of 1 × 10^6^ cm^−1^, our spatial resolution of ~130-nm FWHM corresponds to probing 10 to 15 excitons. This suggests that the CNT may offer an ideal platform to observe and potentially manipulate the IR response at the single-exciton level.

In summary, we investigated local electron-hole dynamics in CNTs using ultrafast IR *s*-SNOM. We demonstrated that ultrafast IR *s*-SNOM registers a local MIR transient with high stability and spatiotemporal resolution, identifying unambiguous heterogeneity despite the lack of any corresponding topographic features in AFM (Fig. 2). Within individual and isolated CNTs, the nonuniformity of electron-hole pair formation is linked to local strain (Fig. 3). In CNT bundles, the observed dynamical heterogeneity is attributed to excitation transfer among tubes, influenced by their composition and spatially evolving tube-tube interactions (Fig. 4). These findings highlight the critical role of local environments in regulating the creation and annihilation of electron-hole pairs, which ultimately determine the optoelectronic properties of CNTs. This behavior is inherently related to their one-dimensional nature and exceptionally high surface-to-volume ratio. Using the previously proposed 1s-2p intra-excitonic transition and a simple model with reasonable parameters, we successfully reproduced the local MIR transient signal amplitudes and the contrasting behavior of ground- and excited-state SNOM profiles (Fig. 5). The theoretical analysis confirms the sensitivity and precision of ultrafast IR nano-imaging in probing excitonic dynamics in one-dimensional systems, corroborating our experimental observations. The local electron-hole dynamics and their transport may be harnessed by integrating CNTs into nanostructured environments. Our demonstrated capability of ultrafast IR nano-imaging will facilitate such efforts by visualizing the local evolution of electron-hole pairs in CNTs.

## MATERIALS AND METHODS

### Experimental design

Further details on the items below can be found in Supplementary Notes 1 and 2.

#### Preparation of CVD-grown single-walled CNTs

Horizontally aligned CNTs were synthesized on r-cut quartz substrates using ethanol as the carbon source. Ti, Pt, and SiO_2_ layers were deposited for alignment markers, and Fe catalysts were evaporated onto patterned regions. Growth occurred at 800°C with ethanol vapor under controlled pressure (1.2 kPa total, 105-Pa ethanol). Substrates were cooled in an Ar/H_2_ atmosphere, and subsequent measurements were performed without transfer.

#### Laser system

A Yb:KGW oscillator (1030 nm, ~150 fs, 76 MHz) was used to pump an optical parametric oscillator, and the following difference frequency generation between the signal and idler pulses generated MIR pulses tunable from 100 to 250 meV. Visible pump pulses at 515 nm were obtained via second harmonic generation of the 1030-nm pulse. Ultrafast IR *s*-SNOM measurements used a 6-μm probe pulse.

#### *Ultrafast IR* s*-SNOM*

The *s*-SNOM setup integrated an AFM, Michelson interferometer, and HgCdTe detector. Gold-coated AFM tips enhanced the mechanical stability of the AFM as well as the optical scattering amplitude. Visible pump pulses (515 nm/2.4 eV) and MIR probe pulses (6 μm/0.21 eV) were illuminated to the sample with variable delay times. The transient MIR signal was detected as a pump-induced change in the scattering of the MIR probe. In standard IR *s*-SNOM, near-field scattering localized at the apex of a metallic AFM tip was extracted by lock-in detection, demodulating the scattering intensity at the harmonics *n*ω_t_ (*n* = 2, 3, …) of the tip tapping frequency ω_t_. This leads to the well-established ground-state near-field scattering amplitude *I_n_* (*69*). In ultrafast IR *s*-SNOM, we selectively detected the near-field pump-probe signal by demodulating the scattering at *n*ω_t_ ± Ω_M_, where Ω_M_ was the modulation frequency of the excitation pulses (*38*). The acquired signal Δ*I_n_* corresponds to the photoinduced change in *I_n_* but is influenced by interference from background scattering, complicating its interpretation (*35*). To remove background interference, we used a two-phase homodyne detection scheme (*38*). By introducing an external reference pulse and adjusting its time delay *t*, we measured the demodulated signal at the maximum (φ_max_) and minimum (φ_min_) points of the interference to extract the field-level pump-probe signal amplitude as Δ*S_n_* = Δ*I_n_*(φ = φ_max_) − Δ*I_n_*(φ = φ_min_). This approach effectively eliminates background effects, and we present Δ*S*_2_ as the near-field pump-probe signal free from the associated complications. While demodulation at higher harmonics (*n* = 3) slightly improves spatial localization, it instead reduces the signal amplitude, limiting its practical utility. This trade-off was validated by our experimental and theoretical analysis (see Supplementary Note 3 and figs. S1 and S2). Thermally induced signals, which are effectively independent of the pump-probe timing because of their slow evolution, were subtracted to extract transient electron-hole pair dynamics by acquiring pump-probe data and imaging at *T* < 0 ps, in which the probe pulse precedes the pump pulse in time (*38*). The thermally induced signals were then substracted from the positive timing data to yield the contrasts purely arising from transient electron-hole dynamics.

#### Far-field reflection pump-probe spectroscopy

Mid-IR probe pulses were tuned across their range, focused noncollinearly with a visible pump pulse. Both pump and probe pulses were *s*-polarized, parallel to the alignment of CNTs. The pump beam diameter was 1.5 to 2 times larger than that of the probe pulse to ensure homogeneous excitation. A relatively small variation in the pump-probe spatial overlap at different probe frequencies was taken into account in the analysis of the pump-probe signal amplitude and its spectral dependence.

#### Raman microscopy

A 532-nm laser was focused as an excitation beam, and scattered light was collected with a charge-coupled device detector. Raman mapping was performed with 0.6-μm intervals, with spectra calibrated against standard reference materials.

#### Peak-force-tapping AFM

Peak-force-tapping AFM (Dimension XR Icon system, Bruker) was performed with triangular silicon nitride tips (SCANASYST-AIR, Bruker). The tapping amplitude was set to 100 nm, and the peak force was maintained at 1 nN.

#### Theoretical approach

The intra-excitonic transitions in CNTs were modeled using an analytical framework to calculate the photoinduced change in the dielectric function associated with the 1s-2p intra-excitonic transition (*64*). The dielectric screening constant and damping rate were optimized so that the ensemble-averaged reflection pump-probe spectrum is best reproduced. To simulate near-field interactions, the CNT was approximated as a dielectric cylinder responding to an external field. The local dielectric response of the CNT was represented by a line dipole density, while the tip apex was modeled by a point dipole centered at a metallic sphere. A self-consistent solution was obtained for the fields and dipoles generated by the tip dipole, segmented CNT dipoles, and their images within the substrate. The polarization induced in the system was calculated for harmonically modulated tip-sample distance with the tapping amplitude of 70 nm to match the experimental condition. The effective tip radius (*R* = 200 nm) was set to reproduce the experimentally observed spatial profile of the near-field pump-probe signal, Δ*S*_2_.

## Supplementary Material

20250618-1

## Supplementary Materials

### This PDF file includes:

Supplementary Notes 1 to 10 Figs. S1 to S10
