# Supplementary material for: Ultrafast infrared nano-imaging of local electron-hole dynamics in CVD-grown single-walled carbon nanotubes
Source: Sci Adv. 2025 Jun 18;11(25):eadv9584. doi: 10.1126/sciadv.adv9584 (PMC12175881; doi:10.1126/sciadv.adv9584)
Supplement: Supplementary file 2 — Supplementary Notes 1 to 10 Figs. S1 to S10 [file sciadv.adv9584_sm.pdf]

Science Advances
AAAS

Supplementary Materials for

Ultrafast infrared nano-imaging of local electron-hole dynamics in CVD-grown single-walled carbon nanotubes

Jun Nishida *et al.*

Corresponding author: Jun Nishida, nishida@ims.ac.jp; Takashi Kumagai, kuma@ims.ac.jp

Sci. Adv. **11**, eadv9584 (2025)
DOI: 10.1126/sciadv.adv9584

This PDF file includes:

Supplementary Notes 1 to 10
Figs. S1 to S10

Supplementary Note 1: Experimental Details

Preparation of CVD-grown single-walled carbon nanotubes (CNTs).

Horizontally aligned single-walled carbon nanotubes (CNTs) are synthesized from ethanol on r-cut single-crystalline quartz substrates (Hoffman Materials Inc.), as described in detail elsewhere (*47*). Ti, Pt, and SiO_2 (1, 25, and 20 nm thick, respectively) were deposited on the substrates in selected regions, which serve as alignment markers to locate the same CNTs for the following measurements. Fe catalysts with a nominal thickness of 0.1 nm were then thermally evaporated in photolithographically patterned stripes. The catalysts were reduced in an Ar atmosphere containing 3% H_2 (~40 kPa, 300 sccm) at 800°C for 10 min. For the growth of CNTs, the flow rate of Ar/H_2 was decreased to 50 sccm, followed by the supply of ethanol vapor as a carbon source. Typical total pressure and ethanol partial pressure were 1.2 kPa and 105 Pa, respectively. After a certain growth duration (typically 4 min), the substrates were cooled to room temperature in an Ar/H_2 atmosphere. The following measurements were conducted on the same substrates without transferring to other substrates.

Laser System

The laser system used in this study is identical to the one described in our recent publication (*38*). A Yb:KGW oscillator (FLINT, Light Conversion) with a central wavelength of 1030 nm, pulse duration of ~150 fs, repetition rate of ~76 MHz, and pulse energy of ~0.12 μJ, serves as the pump source for an optical parametric oscillator (Levante fs, APE GmbH). The signal and idler pulses generated by the optical parametric oscillator undergo difference frequency generation (HarmoniXX DFG, APE GmbH) to produce mid-infrared pulses tunable in the range of 100–250 meV. These pulses have a spectral full-width-at-half-maximum (FWHM) of ~18.5 meV.

For the ultrafast IR *s*-SNOM measurements, the probe frequency was set to ~205 meV. A portion of the output from the Yb:KGW oscillator was also used to generate a 515-nm pump pulse through second harmonic generation in a 1-mm-thick BBO crystal (CASTECH). The resulting visible pump pulse was used for selective excitation of the sample.

Ultrafast infrared scattering scanning near-field optical microscopy (ultrafast IR s-SNOM)

The ultrafast IR *s*-SNOM system employed in this study is based on a commercial nano-FTIR spectrometer (neaSNOM, neaspec GmbH), as described in our recent publication (*38*). The system

integrates an atomic force microscope (AFM), an asymmetric Michelson interferometer, and an HgCdTe detector. The experimental schematics are shown in Figure 1A of the main text.

For the AFM tip, we used a commercially available Pt/Ir-coated silicon tip (ARROW-NCPt, NanoWorld) that was further coated with ~200 nm of gold via sputtering deposition. This additional gold coating enhanced the mechanical stability and optical scattering amplitude of the tip, though it resulted in some reduction in spatial resolution (see Figure S2). The coating also mitigated a long-lasting pump-probe signal likely originating from the silicon core of the tip (see Figure S3). The AFM was operated in tapping mode, with a typical resonance frequency of ~230 kHz and a tapping amplitude of 70–80 nm.

The visible pump pulse (2.4 eV) was focused on the sample, followed by a mid-infrared probe pulse (0.21 eV), with a tunable pump-probe delay time T. A mechanical chopper (MC2000B, Thorlabs) modulated the intensity of the visible pump pulse at a frequency $\Omega_{\mathrm{M}} \approx 10$ kHz. Scattered mid-infrared light from the tip apex was interfered with a reference pulse in the asymmetric Michelson interferometer. The combined signal was detected by the HgCdTe detector, which was coupled with a high-pass filter (EF507, Thorlabs) to eliminate DC and first-harmonic components. The signal was subsequently amplified (DHPVA-101, FEMTO Messtechnik GmbH) and demodulated with a digital lock-in amplifier (HF2LI, Zurich Instruments).

To isolate the near-field pump-probe signal (ΔI_n), the detector output was lock-in demodulated at $n\omega_{\mathrm{AFM}} \pm \Omega_{\mathrm{M}}$ with $n = 2$ or 3. To extract the field-level pump-probe signal ΔS_n of interest, the two-phase homodyne measurements were performed by setting the reference phase to $\varphi = -45°$ and $+135°$ relative to the ground-state scattering S_3 ($\varphi = 0°$). Note that the positive sign corresponds to a further optical delay. The difference of the two measurements $\Delta I_n(\varphi = -45°)$ and $\Delta I_n(\varphi = +135°)$ was regarded as the pump-probe signal. This phase setting gave rise to the maximal signal level, corroborated by that the similar measurement with $\varphi = +45°$ and $-135°$ resulted in much weaker signal amplitude. This interferometric detection scheme was critical for eliminating background scattering, as detailed in the main text and previous works (*38*, *41*).

We also observed a thermally induced signal due to the modulation of the pump excitation. This component should evolve on timescales much longer than 100 ps, manifesting as a time-independent offset. To extract the transient signal associated with electron-hole pair dynamics in CNTs of interest, we subtracted the thermally induced offset, measured at $T < 0$ (where the probe precedes the pump), from the total signal (*38*).

Far-Field Reflection Pump-Probe Spectroscopy

Far-field reflection pump-probe spectroscopy was performed using the same laser system described above, with the mid-infrared probe frequency tuned across its full tunable range. The pump and probe pulses were focused non-collinearly onto the sample at a crossing angle of ~15°. The pump beam was focused using an N-BK7 lens with a focal length of 150 mm, while the mid-infrared beam was focused using a ZnSe lens with a focal length of 100 mm. The sample consisted of high-density CVD-grown carbon nanotubes on r-cut quartz. The sample plane was oriented such that the carbon nanotubes were aligned with the pump and probe polarizations, both of which were *s*-polarized. The probe pulse was incident on the sample at an angle of 45°.

To account for variations in beam size, the pump beam was designed to be significantly larger (Gaussian radius $w \approx 126$ μm) than the probe beam ($w \approx 60$ to 85 μm), ensuring that the probe experienced a uniform pump intensity. A correction factor was applied to compensate for the slight frequency-dependent overlap of the pump and probe beams. This correction enhanced the signal level by up to ~20% on the lower-frequency side compared to the higher-frequency side.

The transient reflection spectra shown in Figure 5C were obtained by averaging data from four independent measurements, all conducted at an excitation fluence of 0.8 μJ/cm^2. Because the probe frequencies varied slightly between measurements, the data was first interpolated with a common frequency base. Additionally, to account for different levels of the pump-probe signals due to the heterogeneous distribution of CNTs in the sample, individual spectra were normalized to the mean signal level between 210 and 250 meV. The normalized spectra were averaged, and the resulting data was re-scaled to retrieve the absolute averaged signal amplitude in the 210–250 meV range. The final transient reflection spectrum is plotted as dots in Figure 5C, with vertical error bars representing one-sigma deviations from averaging and horizontal error bars reflecting the FWHM bandwidth of the mid-infrared spectrum.

Raman microscopy

Raman measurements were performed using either a commercial Raman spectrometer (Renishaw, inVia) or a home-built microscopy system. In the former system, a 532 nm laser expanded into a ~20-µm-long line shape was used to excite the CNTs at a power of ~30 mW through an objective lens with a numerical aperture (NA) of 0.85. Raman spectra were obtained using a grating with 1800 lines/mm. The typical exposure time was 10 s, and the stage was scanned in the tube axial

direction in 0.6 μm steps to form two-dimensional mapping. In the latter system, a diode-pumped solid-state laser (gem 532, Laser Quantum) with a wavelength of 532 nm was used. The excitation laser beam with a linear polarization parallel to the CNT axis was focused onto the sample by an objective lens with a numerical aperture of 0.75 and a focal length of 3.6 mm. Typical laser power was 5 mW, and the temperature rise of CNTs by laser irradiation was negligible. Raman scattering from the sample was collected using the same objective lens, separated by an edge filter (532USLPF-25.0, IRIDIAN Spectral Technologies), and collected by a CCD detector (PIXIS-100BRX, Teledyne Princeton Instruments) mounted to a 300-mm spectrometer (HRS-300, Teledyne Princeton Instruments) with a 1200 lines/mm grating blazed at 500 nm. Raman spectra were calibrated against signals from sulfur, silicon, and naphthalene. Raman mapping measurements were performed by raster scanning the sample mounted on an automated three-dimensional stage at 0.6 μm intervals.

Peak force tapping atomic force microscopy

Peak force tapping atomic force microscopy (AFM) was conducted under ambient conditions using a Dimension XR Icon system (Bruker) at room temperature. The measurements were conducted in a controlled environment to minimize potential disturbances from external factors, such as vibrations or acoustic noise, by placing the AFM instrument inside an acoustic enclosure. A triangular silicon nitride tip (SCANASYST-AIR, Bruker) was used, chosen for its high sensitivity and precision. The tapping amplitude was set to 100 nm, and a peak force setpoint of 1 nN to ensure the probe gently interacts with the surface without causing damage or deformation. The AFM scanner measured the height sensor signals at specific locations where the setpoint force was detected, enabling the acquisition of precise topographic images that reveal fine surface morphology.

Supplementary Note 2: Theoretical Details

Intra-excitonic transition in CNTs

We adapted an analytical formula previously suggested (*64*), which successfully reproduced transient mid-infrared response in a CNT (*44*), to derive the dielectric function for the 1s-2p intra-excitonic transition in a (17, 9) CNT with the diameter of ~1.8 nm (to compare with the far-field ensemble-averaged result in Figure 5C, we instead calculated the excitonic dielectric function for the (14, 4) tube with the diameter of ~1.3 nm; see Supplementary Note 8 and Figure S8 for details). The analytical formula describes the wavefunctions for 1s and 2p states of a bound electron-hole pair on a cylindrical surface, with respect to the electron-hole distance along tube x.

$$\psi_{1\mathrm{s}}(x) = \sqrt{\frac{8}{(a_B^*)^3 \alpha_{1\mathrm{s}}^3 B_{1\mathrm{s}}}}\, |x| \exp\left[-\frac{|x|}{a_B^* \alpha_{1\mathrm{s}}}\right] U\left(1-\alpha_{1\mathrm{s}}, 2, \frac{2|x|}{a_B^* \alpha_{1\mathrm{s}}}\right) \qquad (1)$$

$$\psi_{2\mathrm{p}}(x) = \sqrt{\frac{2}{(a_B^*)^3}}\, x \exp\left[-\frac{|x|}{a_B^*}\right] \qquad (2)$$

Here, the effective Bohr radius, a_B^*, is determined by the reduced mass μ of the exciton and the effective permittivity ϵ of the surrounding environment.

$$a_B^* = \frac{4\pi\epsilon\epsilon_0 \hbar^2}{\mu e^2} \qquad (3)$$

$U(a,b,z)$ is Kummer's confluent hypergeometric function of the second kind, and $B_{1\mathrm{s}}$ is derived from the function as the following:

$$B_{1\mathrm{s}} = 2\int_0^\infty y^2 e^{-y}\left[U(1-\alpha_{1\mathrm{s}}, 2, y)\right]^2 dy \qquad (4)$$

In addition, $\alpha_{1\mathrm{s}}$ as a dimension-less parameter is derived numerically by solving the following equation.

$$\ln(\alpha_{1\mathrm{s}}) - \Psi(1-\alpha_{1\mathrm{s}}) - \frac{1}{2\alpha_{1\mathrm{s}}} = \ln\left(\frac{r_{\mathrm{CNT}}}{a_B^*}\right) - 2\Psi(1) \qquad (5)$$

where $\Psi(x)$ is the digamma function, and r_{CNT} is the radius of the CNT of interest. The quantized energy levels for the 1s and 2p excitonic states are derived as

$$E_{1\mathrm{s}} = -\frac{\hbar^2}{2\mu\left(\alpha_{1\mathrm{s}} a_B^*\right)^2} \qquad (6)$$

$$E_{2p} = -\frac{\hbar^2}{2\mu\left(a_B^*\right)^2} \qquad (7)$$

The oscillator strength, which characterizes the strength of the optical transition between the 1s and 2p states, was calculated using the derived wavefunctions and is given by

$$f = \frac{128\mu\left(a_B^*\right)^2(\alpha_{1s})^5}{\hbar^2 B_{1s}}(E_{2p} - E_{1s}) \times \left(\int_0^\infty s^3 e^{-s(1+\alpha_{1s})}\, U(1-\alpha_{1s}, 2, 2s)\, ds\right)^2 \qquad (8)$$

and then constructed the exciton-induced dielectric function as (*45*)

$$\Delta\epsilon = \frac{n_{ex}e^2}{\epsilon_0\mu}\frac{f}{\left(\omega_{2p} - \omega_{1s}\right)^2 - \omega^2 - i\omega\Gamma} \qquad (9)$$

where ω_{2p} and ω_{1s} are the angular frequencies corresponding to the energies in eq. (7) and (6), n_{ex} is the three-dimensional density of the exciton, and Γ is the dephasing of the transition.

We determined the effective reduced mass μ based on the previously provided formula for the lowest state (E_{11}) exciton (*65*), leading to $\mu = 0.027m_0$ with the free electron mass m_0. Also, we set the effective permittivity ϵ and the dephasing Γ to 5 and 90 meV, respectively, to reproduce the experimental far-field pump-probe spectral profile. These values are fairly close to the previously inferred values of 6 and ~100 meV (*44*). These together led to the derived oscillator strength of $f = 0.43$. By converting the exciton density of $n_{1D} = 1 \times 10^6$ cm^{-1} to the three-dimensional equivalent based on the radius of the tube, we derived the on-axis dielectric function of the carbon nanotube as shown in Figure 5B in the main text.

Dielectric cylinder in a uniform field

As one of the steps to understand the near-field interaction between a CNT and the tip apex, we model the CNT as an infinitely long dielectric cylinder and consider how it responds to an externally applied uniform field. In the Cartesian coordinate (*x, y, z*), the dielectric cylinder is oriented along the *z*-axis, with the uniform field applied along the *x*-axis, perpendicular to the cylinder axis. In the cylindrical coordinate (*r*, *φ*, *z*), the potentials outside and inside the dielectric cylinder are given by

$$\Phi_{out} = -E_0 r\cos\varphi + \beta_{CNT}R^2 E_0\frac{\cos\varphi}{r} \qquad (10)$$

$$\Phi_{in} = -\frac{2}{\epsilon_{CNT}+1}E_0 r\cos\varphi \qquad (11)$$

where R and ϵ_{CNT} are the radius and the dielectric function of the CNT, and E_0 is the magnitude of the applied external field. β_{CNT} is given by

$$\beta_{\text{CNT}} = \frac{\epsilon_{\text{CNT}} - 1}{\epsilon_{\text{CNT}} + 1} \qquad (12)$$

The potentials in eq. (10) and (11) satisfy Laplace's equation ($\nabla^2\Phi = 0$) and the boundary conditions at the cylinder surface ($\Phi_{\text{out}} = \Phi_{\text{in}}$ and $\partial\Phi_{\text{out}}/\partial r = \epsilon_{\text{CNT}}\partial\Phi_{\text{in}}/\partial r$ at $r = R$).

We focus on the potential outside the dielectric cylinder in eq. (10). The first term ($-E_0 r \cos\varphi$) originates from the applied external field. The second term arises from the dielectric response of the CNT against the applied field. The meaning of the second term is evident by considering the potential around a one-dimensional dipole line density p_{line}, where the dipole is oriented parallel to the x-axis and the line density axis is oriented along the z-axis. For such a line density, the effective dipole at a fraction of the length dz is given by $p_{\text{line}}dz$. The potential surrounding the one-dimensional dipole density is given by

$$\Phi_{\text{line}} = \frac{p_{\text{line}}}{2\pi\epsilon_0}\frac{\cos\varphi}{r} \qquad (13)$$

By comparing the second term in eq. (10) and eq. (13), we find that the response of the dielectric cylinder is equivalent to the one-dimensional dipole line density of

$$p_{\text{line}} \equiv \alpha_{\text{line}}E_0 = [2\pi\epsilon_0 R^2 \beta_{\text{CNT}}]E_0 \qquad (14)$$

where [...] corresponds to the line polarizability α_{line} of the dielectric cylinder, giving rise to eq. (1) in the main text.

Near-field interactions between the tip apex and the one-dimensional cylinder

Based on eq. (14), we address the near-field interaction between the tip apex and the CNT on a quartz substrate. We regard the CNT as a dielectric cylinder which is oriented along x-direction, and the surface normal and the applied field are along z-direction (Figure 5D, main text). We first consider the case where the tip apex is centered on the CNT. We represent the tip apex as a metallic sphere of radius R_{sph} and further approximate its near-field interaction by a point dipole located at the center (*69*).

When the tip experiences a uniform external field $E_{\text{ext,sph}}$, the induced dipole moment in the tip can be expressed as:

$$p_{\text{tip}} \equiv \alpha_{\text{tip}}E_{\text{ext,sph}} = [4\pi\epsilon_0 R_{\text{sph}}^3]E_{\text{ext,sph}} \qquad (15)$$

The tip dipole creates a non-uniform field around the CNT. However, due to the exceedingly small diameter of the CNT relative to the tip radius, the field experienced by the CNT locally can be approximated as uniform. Therefore, the CNT is divided to N segments (D_1, D_2, …, D_N) of length Δx, each experiencing a field $E_{\mathrm{ext},i}$ (i = 1, 2, …, N) perpendicular to its axis. The induced point dipole at each segment is then given by

$$p_{\mathrm{CNT},i} \equiv \alpha_{\mathrm{CNT}} E_{\mathrm{ext},i} = [2\pi\epsilon_0 R^2 \beta_{\mathrm{CNT}} \Delta x] E_{\mathrm{ext},i} \quad (i = 1, 2, \dots, N) \qquad (16)$$

Due to the presence of the quartz substrate, image dipoles are formed for both the tip and the CNT segments. For a real dipole p_{real} above the substrate, the corresponding image dipole is formed at the mirrored location across the surface, and its amplitude is given by $p_{\mathrm{image}} = \beta_{\mathrm{sub}} p_{\mathrm{real}}$ with $\beta_{\mathrm{sub}} = (\epsilon_{\mathrm{quartz}} - 1)/(\epsilon_{\mathrm{quartz}} + 1)$, where $\epsilon_{\mathrm{quartz}}$ is the dielectric function of quartz.

We now seek a self-consistent solution by tracking field interactions among the tip dipole p_{tip}, the N CNT dipoles $p_{\mathrm{CNT},i}$ (from the segmented domains), the tip image dipole $p_{\mathrm{tip,image}}$, and the N CNT image dipoles $p_{\mathrm{CNT},i,\mathrm{image}}$, by focusing on the z-component of the field. Here, we define $G_{X\to Y}$ as a factor relating the dipole at the location X to the z-component of the field it forms at the location Y (*i.e.*, $E_Y = G_{X\to Y} p_X$). The tip dipole induced by the laser field (E_0) and the fields from the other dipoles is then given by

$$\begin{aligned} p_{\mathrm{tip}} &= \alpha_{\mathrm{tip}} \left[E_0 + \sum_j (G_{\mathrm{CNT},j\to\mathrm{tip}})(p_{\mathrm{CNT},j}) + \sum_j (G_{\mathrm{CNT,image},j\to\mathrm{tip}})(p_{\mathrm{CNT,image},j}) + (G_{\mathrm{tip,image}\to\mathrm{tip}})(p_{\mathrm{tip,image}}) \right] \\ &= \alpha_{\mathrm{tip}} \left[E_0 + \sum_j (G_{\mathrm{CNT},j\to\mathrm{tip}} + \beta_{\mathrm{sub}} G_{\mathrm{CNT,image},j\to\mathrm{tip}})(p_{\mathrm{CNT},j}) + (\beta_{\mathrm{sub}} G_{\mathrm{tip,image}\to\mathrm{tip}})(p_{\mathrm{tip}}) \right] \end{aligned} \qquad (17)$$

Each segment within the CNT is also polarized by the laser field and the image dipoles:

$$\begin{aligned} p_{\mathrm{CNT},i} &= \alpha_{\mathrm{CNT}} \left[E_0 + (G_{\mathrm{tip}\to\mathrm{CNT},i})(p_{\mathrm{tip}}) + \sum_j (G_{\mathrm{CNT,image},j\to\mathrm{CNT},i})(p_{\mathrm{CNT,image},j}) + (G_{\mathrm{tip,image}\to\mathrm{CNT},i})(p_{\mathrm{tip,image}}) \right] \\ &= \alpha_{\mathrm{CNT}} \left[E_0 + (G_{\mathrm{tip}\to\mathrm{CNT},i} + \beta_{\mathrm{sub}} G_{\mathrm{tip,image}\to\mathrm{CNT},i})(p_{\mathrm{tip}}) + \sum_j (\beta_{\mathrm{sub}} G_{\mathrm{CNT,image},j\to\mathrm{CNT},i})(p_{\mathrm{CNT},j}) \right] \end{aligned} \qquad (18)$$

The N+1 simultaneous linear equations in eqs. (17) and (18) are solved to derive p_{tip} and $p_{\mathrm{CNT},i}$, and the sum of these dipole ($P_{\mathrm{tot}} = p_{\mathrm{tip}} + \sum_j p_{\mathrm{CNT},j}$) is regarded as the polarization source giving rise to the scattering.

As noted in the main text, we harmonically modulate the tip–sample distance by $H = A[1 + \cos 2\pi t]$ and the resulting polarization is demodulated by $\cos 2\pi nt$ to yield the scattering profile S_n. The same procedure is repeated by switching the CNT dielectric function ϵ_{CNT} to that of the excited state, and the deviation from the original response, ΔS_n, is regarded as the pump-induced change in the demodulated signal that is directly comparable to the experimental observables. We also performed the same calculation with the tip dipole laterally offset from the carbon nanotube, which led to the simulation of the imaging as in Figure 5E.

Upon the simulation, the CNT of the total length of 3200 nm was divided into 860 segments with 2 nm step. The radius of the CNT was set to 0.9 nm, while the tip radius of 200 nm was employed. The ground-state refractive index of the CNT was assumed to be ($\tilde{n} = 1.4 + i1.4$) (*67*) and the tapping amplitude of A = 70 nm was used for the harmonic modulation of the tip-sample distance.

Supplementary Note 3: Spatial Resolutions from Experiment and Theory

Experimental spatial resolution

To evaluate the experimental spatial resolution of our ultrafast IR *s*-SNOM measurements, we analyze the topography and the near-field pump-probe signals ΔS_2 and ΔS_3, acquired simultaneously at a pump-probe delay of $T = 0$ ps (Figure S1a). The lateral profiles of the near-field pump-probe signals for ΔS_2 and ΔS_3 were extracted and fitted to Gaussian functions, yielding full-width-at-half-maximum (FWHM) values of 130 nm for ΔS_2 and 110 nm for ΔS_3 (Figure S1b). These results confirm that the third harmonic signal provides slightly better spatial resolution, albeit at the cost of reduced signal amplitude. In Figure S1c, the spatial decay is plotted over a larger range up to 300 nm, as a function of the distance from the tube location (Δx). The exponential decay length scales (70 nm for ΔS_2 and 60 nm for ΔS_3) are reasonable given the quartz substrate with weak dielectric response. The relatively minor offset likely arises from the combination of further global structures of the tip, as well as the pump-probe signal from the tip itself as discussed in Supplementary Note 4 and Figure S3.

The only modest improvement in spatial resolution in ΔS_3 over ΔS_2 is attributed to the dielectric nature of the quartz substrate used in this study, instead of metallic substrates. The dielectric substrate likely suppresses strong harmonic dependence, as supported by theoretical simulations (Figure S2). Additionally, the gold coating, applied to the tip to enhance mechanical stability and

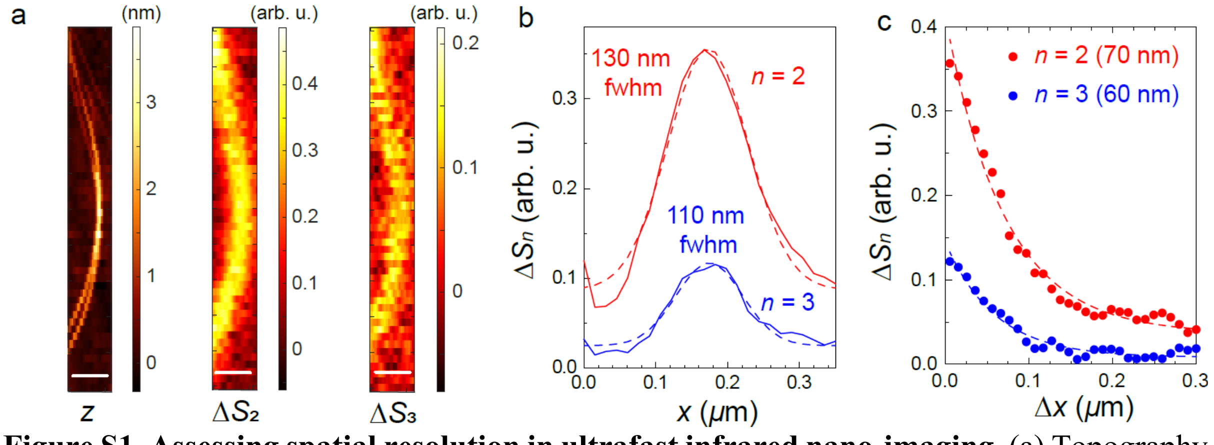

Figure S1. Assessing spatial resolution in ultrafast infrared nano-imaging. (a) Topography (left), ΔS_2 (middle), and ΔS_3 (right) imaging simultaneously acquired at the pump-probe delay of $T = 0$ ps. Scale bar – 200 nm. (b) Extracted lateral profile of the near-field pump-probe signal for $n = 2$ and $n = 3$. (c) The spatial decay of the pump-probe signal over longer length scales up to 300 nm, plotted with respect to the distance from the tube position Δx.

scattering amplitude, may have increased the effective tip radius, further limiting the spatial resolution.

Despite these limitations, the achieved spatial resolution is sufficient to resolve the key heterogeneities within the CNTs, as discussed in the main text. The trade-off between signal intensity and resolution suggests that ΔS_2 is more advantageous for quantitative analysis in this work, given the relatively small gain in resolution provided by ΔS_3.

Theoretical spatial resolution

In Figure S2, we present the theoretically calculated lateral profile of the near-field pump-probe signal. The spatial localization of the signal is strongly influenced by the tip radius (R) and tapping amplitude (A). For the calculations shown, we used $R = 200$ nm and $A = 70$ nm. The magnitude of the normalized signal ($|\Delta S_n/S_n|$) increases with higher harmonic orders (n), accompanied by improved spatial localization as quantified by the FWHM of Gaussian fits to the lateral profiles. Comparing the localization for $n = 3$ and $n = 2$, the localization is improved by approximately 20%, consistent with the experimental observations shown in Figure S1b. However, this modest improvement in localization comes at the cost of a significant reduction in the absolute signal amplitude. Consequently, prioritizing the analysis of ΔS_2 over ΔS_3 is justified in this work, as it provides a better balance between signal strength and spatial resolution.

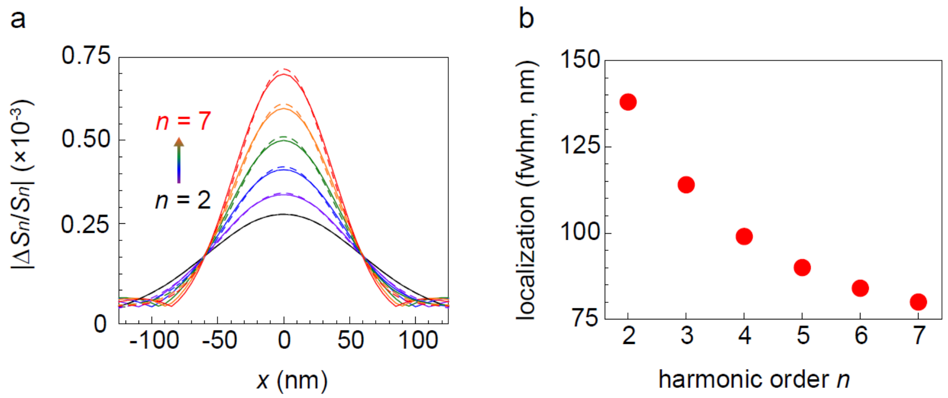

Figure S2. Lateral profile of near-field pump-probe signal based on simulations. (a) Lateral profiles of $|\Delta S_n/S_n|$ for different demodulation harmonics n. Solid line – simulated data, dotted line – fit to Gaussian. (b) Extracted full-width-half-maximum linewidth plotted against demodulation harmonics.

Finally, we note that the effective tip radius R was set to 200 nm so that the calculated spatial profile of ΔS_2 agrees with the experimentally observed profile. As noted above, this choice of R simultaneously reproduces the spatial localization for ΔS_2 and ΔS_3. This effective R likely significantly deviates from the actual radius of the tip apex. This is due to the nature of the point dipole model, which approximates the tip apex as a sphere and disregards its vertically elongated nature. Indeed, it has been suggested that, for a given tip radius, the point dipole model tends to over localize the field at the tip apex compared with, e.g., finite dipole model taking into account the vertical elongation (*70*). Therefore, it is reasonable that the relatively large value of the effective tip radius is needed here to match the experimentally observed localization.

Supplementary Note 4: More on a Correlation between Pump-Probe and Local Strain

Tube with homogeneous G-band intensity

In Figure S3, we analyze a CNT exhibiting a relatively uniform G-band intensity profile. The tube circled by the white dotted line in Figure S3a was selected for detailed analysis. Despite the lack of significant variations in G-band intensity, the corresponding spectral analysis (Figure S3b) reveals substantial modulation in the G-band frequency, indicating the presence of strain variations along the tube.

Interestingly, the absence of notable G-band intensity modulation suggests that the strain is not strongly coupled to the electronic state and associated resonance Raman enhancement mechanism for this tube. The observation is reasonable provided that strain modulation affects electronic states differently depending on the chirality of the CNT (*50*).

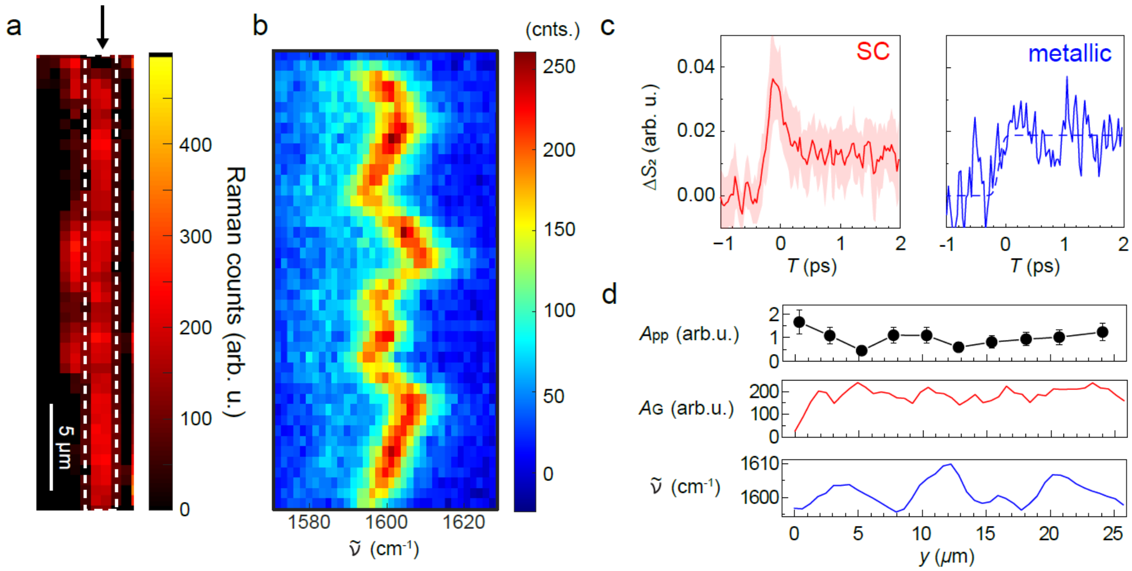

Figure S3. Tube with homogeneous G-band intensity profile. (a) G-band intensity profile of one of the CNTs. The CNT circled by white dotted line is studied in detail. (b) The corresponding evolution of the G-band peak, which demonstrates a large frequency variation despite the lack of major intensity variation. (c) Left – the average of ten pump-probe data sets acquired on the tube at different locations. Right – pump-probe acquired on another metallic CNT. (d) The spatial evolution of the near-field pump-probe signal A_{pp}, G-band intensity A_{G}, and the G-band peak position $\tilde{\nu}_G$.

The near-field pump-probe measurements conducted along the tube show a relatively homogeneous signal, consistent with the uniform G-band intensity (Figure S3d). Averaging multiple pump-probe measurements across the tube reveals a decay profile characterized by a rapid initial component followed by a slower offset-like evolution (Figure S3c, left). This decay profile is plotted in Figure 3B (right) in the main text.

In contrast, we observed a pure offset signal when the pump-probe measurements were performed on a metallic CNT (Figure S3c, right). This suggests that the offset component is unrelated to exciton formation and is instead associated with the tip itself, as confirmed by the measurement on a substrate which gives rise to an essentially identical offset signal. We note that this offset signal is only apparent under the very strong excitation fluence (~300 μJ/cm^2) at the very small signal level ($|\Delta S_n/S_n| < 5 \times 10^{-4}$), rendering this contribution negligible for most applications of ultrafast IR *s*-SNOM.

<u>*Lower fluence measurement*</u>

We performed a similar measurement to the one in Figure 3 of the main text on a different CNT using a lower excitation fluence (~60 μJ/cm^2), as shown in Figure S4. The results replicate the key evolutions and correlations observed in Figure 3, confirming the versatility of the observed trend.

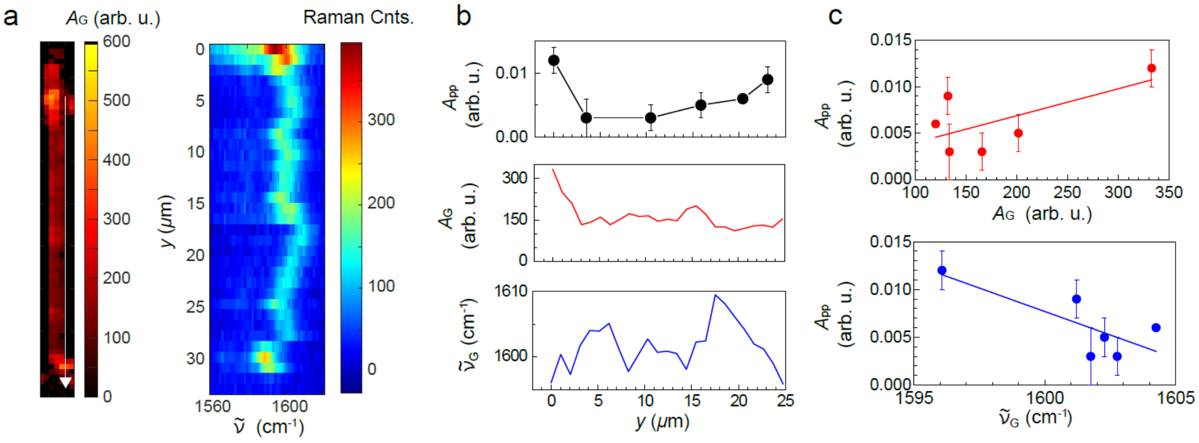

Figure S4. Correlation between pump-probe and local strain with lower pump fluence. (a) G-band intensity profile (left) and spectral evolution (right). (b) The evolution of the near-field pump-probe signal A_{pp} (top), the G-band Raman intensity A_G (middle), and the G-band peak position $\tilde{\nu}_G$ (bottom). (c) The correlation plots between the near-field pump-probe signal amplitude A_{pp} and G-band Raman intensity A_G (middle) or G-band peak position $\tilde{\nu}_G$ (bottom).

Supplementary Note 5: Excitation Fluence Dependence

In Figure S5a, we present near-field pump-probe decay profiles measured on a CNT bundle under varying excitation fluences. The data were fitted to single exponential functions, and the extracted amplitudes and decay time constants are shown in Figures S5b and S5c, respectively. The pump-probe signal amplitude exhibits clear saturation behavior, indicating that the fluence range corresponds to a regime where interactions between electron-hole pairs become significant. Interestingly, the decay time constants appear to be relatively insensitive to excitation fluence. At the high fluence, the exciton wavefunctions overlap significantly and thus suppress the formation of electron-hole pairs as observed in Figure S5b. Then, the high-density electron-hole pairs undergo exciton-exciton annihilation in a reaction-limited regime, leading to the relative insensitivity of the lifetime on the fluence.

Another possible explanation is related to the measurement being performed on a CNT bundle. In this case, electron-hole pairs may transfer to a metallic CNT within the bundle, which acts as a quenching site. This mechanism aligns well with the observed fluence-independent behavior of the decay time constants, because it is the transport of electron-hole pairs to the metallic tube that limits the annihilation. Additionally, the relatively weak dependence of the time constants on fluence may be simply obscured by noise in the current dataset, which is more clearly observed in far-field pump-probe measurements (see Supplementary Note 6 and Figure S6).

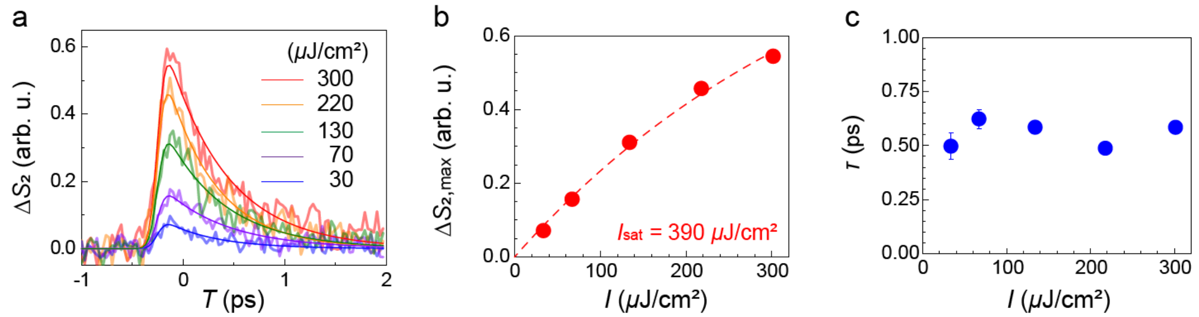

Figure S5. Near-field pump-probe with different excitation fluence. (a) The near-field pump-probe profiles acquired with different excitation fluence. (b) The pump-probe signal amplitudes. Dotted line – fitting to saturation function $A(1\text{-}\exp[-I/I_{\mathrm{sat}}])$. (c) Extracted time constants from the fitting.

Supplementary Note 6: More on a Far-Field Pump-Probe

Data fitting procedure for transient reflection pump-probe spectrum

To model the transient reflection pump-probe spectrum presented in Figure 5C, we assumed a three-layer system consisting of (1) air ($\epsilon_1 = 1$), (2) a sparse CNT layer ($\epsilon_2 = 1 + \Delta\epsilon_{\text{CNT}}$), and (3) a quartz substrate (ϵ_3 with strong spectral dependence due to optical phonon contributions (*68*)). The CNT layer is assumed to be $d = 1.3$ nm thick to represent the average CNT height in the sample. For the ground state, $\Delta\epsilon_{\text{CNT}}$ is set to zero, and for the excited state, this was modeled using either a Lorentzian or Drude response.

The field-level reflection coefficient for the three-layer system is given by

$$r = \frac{r_{12} + r_{23}e^{2i\delta}}{1 + r_{12}r_{23}e^{2i\delta}} \qquad (19)$$

where δ is given by $\delta = k_2 d$, and the Fresnel coefficients r_{ij} are given by

$$r_{ij} = \frac{k_i\epsilon_j - k_j\epsilon_i}{k_i\epsilon_j + k_j\epsilon_i} \qquad (20)$$

with

$$k_i = \frac{2\pi}{\lambda}\sqrt{\epsilon_i - \sin^2\theta_0} \qquad (21)$$

Here, λ is the wavelength, and θ_0 is the incident angle between the top and middle layers. Based on eq. (19), the reflected intensity is calculated as

$$R = |r|^2 \qquad (22)$$

For the transient reflection spectrum, the reflection intensities in eq. (22) for the ground-state (R_{gs}) and the excited state (R_{ex}) were calculated, and the transient response was obtained as

$$\frac{\Delta R}{R} = \frac{R_{\text{ex}} - R_{\text{gs}}}{R_{\text{gs}}} \qquad (23)$$

To account for the finite spectral bandwidth of the mid-infrared probe pulse in the experiment, the calculated spectrum was convoluted with a Gaussian window of 19 meV full-width-at-half-maximum (FWHM). The best fit to the experimental data was achieved with a Lorentzian response characterized by a resonant energy (ω_0) of 139 meV and a damping rate (Γ) of 88 meV. Alternatively, a Drude model provided the best fit with a damping rate (Γ) of 294 meV.

The strong dispersive features observed in the transient reflection spectrum primarily arise from the quartz phonon mode, which was incorporated into the model using literature values for the

dielectric function of quartz (*68*). However, this phonon mode obscures finer spectral details, making it challenging to unambiguously determine the origin of the mid-infrared transient solely based on spectral profiles. Nonetheless, the intra-excitonic transition of bound excitons provides a reasonable explanation for the observed transient response strength in nano-localized pump-probe measurements.

Decay profile in the far-field reflection pump-probe measurements

In Figure S6a, we present the normalized pump-probe decay profiles measured at a probe energy of 205 meV under varying excitation fluences. The data were fitted to single exponential decay functions, with the extracted amplitudes and decay time constants shown in Figures S6b and S6c, respectively. The decay time constants, which exceed 1 ps, are notably slower than those observed in the near-field pump-probe measurements. This discrepancy is attributed to the significantly lower excitation fluence used in the far-field measurements, which is more than an order of magnitude smaller than that in near-field experiments. At higher excitation fluences, exciton-exciton annihilation is more pronounced, resulting in faster decay dynamics in the near-field measurements.

Even under the low-fluence regime of the far-field experiments, the decay time constants exhibit a weak but noticeable dependence on fluence, varying by approximately 50% over two orders of magnitude in fluence (Figure S6c). Interestingly, when extrapolated to higher fluences

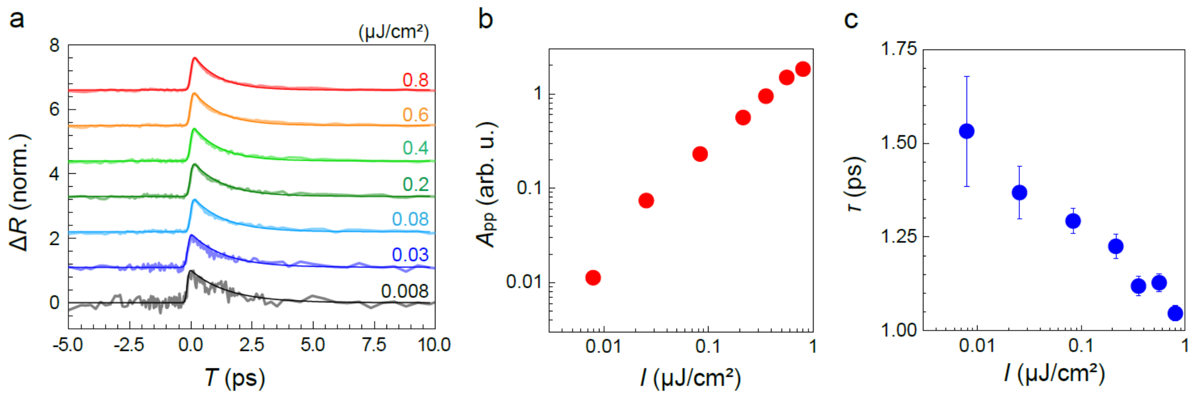

Figure S6. Far-field reflection pump-probe decay profiles. (a) Transparent line – normalized pump-probe decay profiles acquired with different pump fluence, with the probe energy of 205 meV. Solid line – single exponential fits. (b) Extracted fluence dependence of the pump-probe amplitudes. (c) Extracted time constants of the pump-probe decays.

($\sim$100 μJ/cm^2), the decay time constants align well with those measured in the near-field regime (0.3–0.5 ps, Figure S7). This consistency underscores the role of interactions among excitons, which dominate the dynamics at higher fluences. The pump-probe signal amplitudes (Figure S6b) also display saturation behavior at higher fluences, further highlighting the influence of interacting excitons. While the presence of CNT bundles in the sample complicates quantitative interpretation of this ensemble-averaged measurement, these results reflect the established picture of electron-hole dynamics in CNTs, where exciton-exciton annihilation plays significant roles.

Supplementary Note 7: Variation of Near-Field Pump–Probe Dynamics Across CNTs

In Figure S7, we show the near-field pump-probe measurements performed on CNTs from the same sample batch and using the same tip as in Figure 2 in the main text. As depicted in Figure S7a, the pump-probe decay profiles and amplitudes exhibit significant variation from tube to tube. These differences in decay dynamics are more evident in the normalized plots shown in Figure S7b.

Each decay profile in Figure S7a was fitted to a single exponential, and the extracted pump-probe amplitudes and decay time constants were correlated with the apparent topographic heights, as summarized in Figure S7c. Unlike the topographic measurements in Figures 3 and 4 of the main text, which were obtained in peak-force tapping mode, the topography here was measured using standard tapping mode AFM coupled with the *s*-SNOM system. This approach generally underestimates the actual topographic height, leading to smaller measured values. Consequently, tubes with topographic heights between 1 and 2 nm may include bundles rather than single, isolated CNTs.

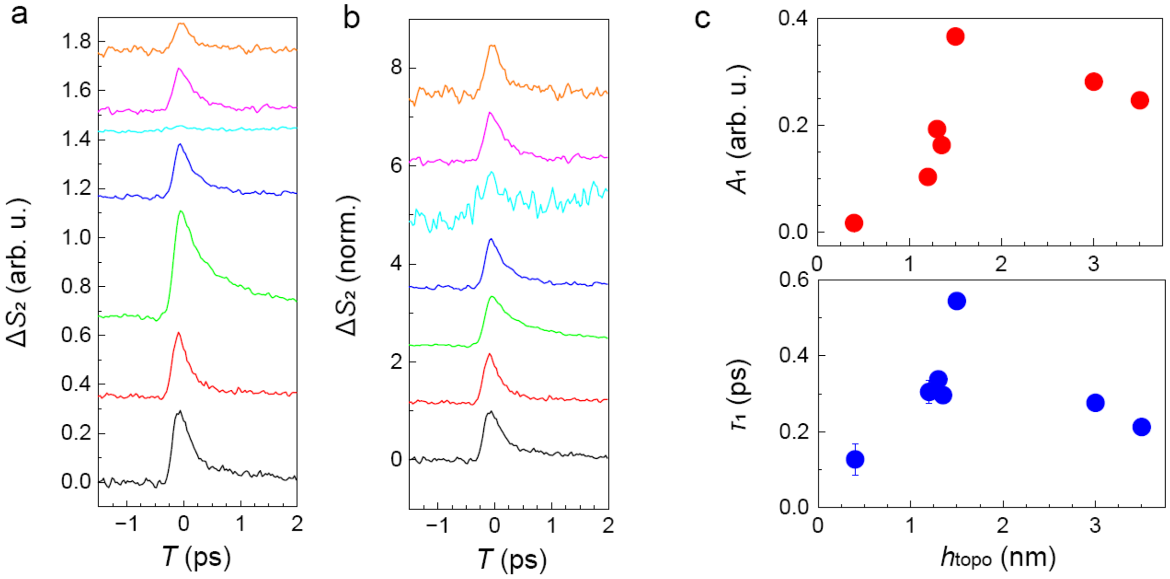

Figure S7. Near-field pump-probe measurements on different tubes. (a) Near-field pump-probe measurements on seven different tubes. (b) Corresponding normalized decays. (c) The correlation plots between the apparent topographic height h_{topo} and the pump-probe signal amplitudes A_1 (top) or decay constants τ_1 (bottom) extracted from the single exponential fits.

The analysis reveals no strong correlation between the pump-probe signal amplitudes or decay time constants and the apparent topographic height. This suggests that topographic variations, and their associated effects on near-field interactions, do not significantly influence the formation or annihilation of electron-hole pairs. However, the pump-probe amplitude appears to saturate at h_{topo} ~ 1.5 nm, and the decay time constants also peak near this height. This behavior may be attributed to a larger number of CNTs on a thicker bundle, which are more likely to contain metallic CNTs. These metallic CNTs may act as quenching sites, suppressing exciton formation and promoting faster recombination of electron–hole pairs.

Supplementary Note 8: Excitonic Dielectric Function for (14, 4) CNT

As noted in the main text and Supplementary Note 2, the model used to reproduce the ensemble-averaged reflection pump–probe spectrum in Figure 4C (main text) was based on a (14,4) CNT. This choice was motivated by its diameter which agrees well with the average CNT diameter in the sample (1.3 to 1.4 nm). We assumed the same dielectric constant ($\epsilon = 5$) and damping rate (Γ = 90 meV) used in Supplementary Note 2. The resulting excitonic wavefunction and associated dielectric function are given in Figure S8. Note that the amplitude of the dielectric function is further scaled to reproduce the far-field pump-probe spectrum in Figure 5C, due to the significantly weaker excitation fluence in the far-field pump-probe experiment (< 1 μJ/cm^2 compared to > 60 μJ/cm^2 in the near-field experiment) and sparsely distributed CNTs within the sample.

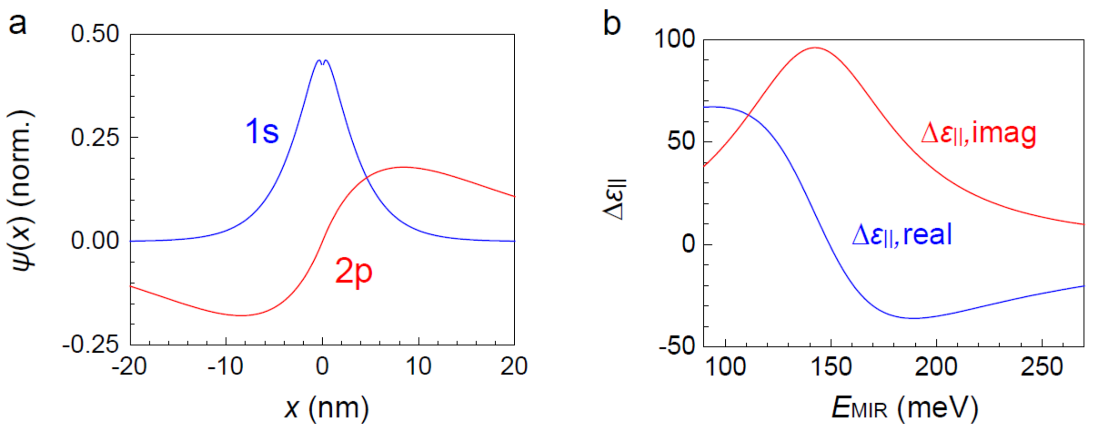

Figure S8. Excitonic wavefunctions and dielectric functions for (14, 4) CNT. (a) Wavefunctions for 1s and 2p states with respect to the relative coordinate between electron and hole, x. (b) Dielectric function calculated assuming the density of 1×10^6 cm^{-1}. Note that the amplitude of the dielectric function was further scaled to reproduce the far-field reflection pump-probe spectrum.

Supplementary Note 9: Quantitative relationship among strain, resonance Raman intensity, and pump-probe signal

In Figure 3D of the main text, as well as in Figure S4, we demonstrate the G-band intensities and the pump-probe signals are correlated positively, whereas the Raman peak positions are anti-correlated with the pump-probe signals. In the main text, we propose a mechanism in which the compressive strain induces the reduction in the E_{44} transition energy, thereby detuning the system from resonance conditions for both Raman and pump-probe excitation. Below, we provide a simple calculation on how this mechanism quantitatively gives correlations between the pump-probe signal and Raman observations.

The shift in Raman G-band peak position is believed to be linearly dependent on the strain imposed on the CNTs (*48*). The impact of the strain on the electronic band structure, particularly on the optical transition energy E_{kk}, is dependent on the chirality (n, m) of the CNT and the specific optical transition of interest. A theoretical relationship between the strain ε and E_{kk} was proposed (*49*), which was later modified to yield a better agreement with an experiment (*50*). The formula is given by (*50*)

$$\frac{E_{kk}}{\varepsilon} = 0.57\mathrm{sgn}(2p+1)(-1)^{k+1}(1+\upsilon)3t_0\cos(3\theta) \qquad (24)$$

where $p \equiv n - m \equiv -1$, 0, or 1 (mod 3), $t_0 = 2.7$ eV, $\upsilon = 0.2$, and θ is the chiral angle. For the E_{44} transition of the (17, 9) tube explored in Figure 3 in the main text, $p = -1$ and eq. (24) yields a positive value. Under compressive strain ($\varepsilon < 0$), eq. (24) predicts the reduction in the energy involved in the E_{44} transition. Assuming a strain-free optical transition energy $E_{44,0} = 2.315$ eV and a chiral angle $\theta = 19.9^\circ$ for the (17, 9) CNT, we compute the strain dependence of the E_{44} energy, as shown in Figure S9a.

Both our Raman excitation energy E_{Raman} (2.33 eV) and pump excitation energy E_{pump} (2.41 eV) are above $E_{44,0}$ and can be considered narrowband compared to the absorption linewidth of the E_{44} transition. Assuming a Lorentzian absorption lineshape with 150 meV fwhm, and that both the resonance Raman signal intensity (A_{G}) and the pump-probe signal (A_{pp}) are proportional to the absorption at each excitation energy, we calculate how A_{G} and A_{pp} depend on the compressive stress up to 1% (Figure S9b). Then, the resulting correlation between A_{G} and A_{pp} demonstrates a nearly linear relationship as shown in Figure S9c.

While experimentally observed correlation between the Raman peak position $\tilde{\nu}_G$ and the pump-probe signal A_{pp} appears to be linear as predicted from eq. (24) and Figure S9b, A_G–A_{pp} correlation may appear nonlinear (Figure 3D, top). This deviation may be due to several factors not accounted for in the simplified model above, including saturation effects of the pump-probe signal as demonstrated in Figure S5, potential strain-induced change in the oscillator strength of the E_{44} transition, as well as non-unimodal excitation-energy dependence of resonance Raman enhancement factor (*52*).

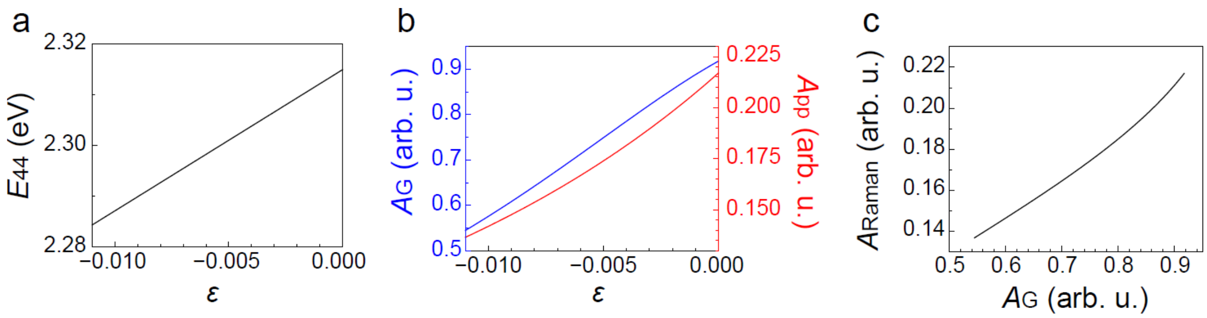

Figure S9. Quantitative analysis of the impact of the strain. (a) The influence of the strain ε on the transition energy E_{44} of (17, 9) tube. (b) Corresponding dependency of resonance Raman intensity A_G (blue) and pump-probe intensity A_{pp} (red) on the strain. (c) The correlation between A_G and A_{pp}, which is approximately linear.

Supplementary Note 10: Ultrafast nano-imaging with low fluence excitation

In Figure S10, we present ultrafast nano-imaging of a CNT bundle performed at a relatively low pump fluence of ~30 μJ/cm². Despite the low pump fluence and the associated noise level, the measurement clearly resolves the transient mid-infrared response and reveals spatial heterogeneity. Notably, a diminished pump–probe response is observed at the center of the image. This non-uniformity is likely associated with the interaction between the CNT bundle and a nearby contaminant particle, as visible in the corresponding topographic image.

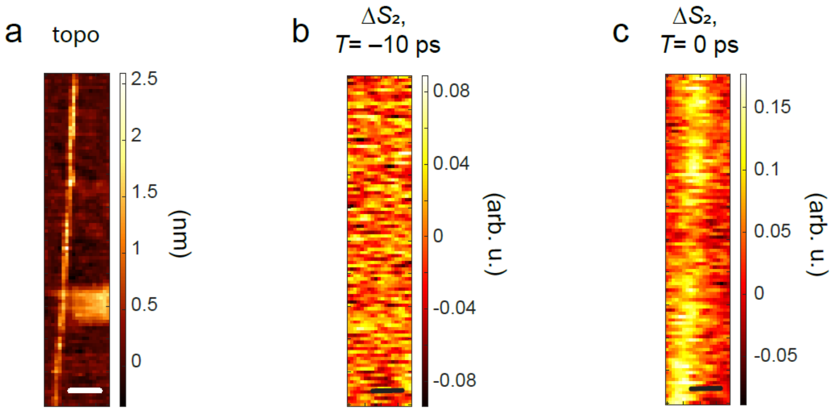

Figure S10. Ultrafast nano-imaging under low excitation fluence. (a) AFM topography of a CNT bundle. (b) Pump–probe nano-imaging at a negative delay ($T = -10$ ps). (c) Pump–probe nano-imaging at zero delay ($T = 0$ ps), revealing signal heterogeneity at the center of the image. Scale bar – 200 nm.
